# Proteomic comparison of *Ralstonia solanacearum* strains reveals temperature dependent virulence factors

**DOI:** 10.1186/1471-2164-15-280

**Published:** 2014-04-12

**Authors:** Ana M Bocsanczy, Ute CM Achenbach, Arianna Mangravita-Novo, Marjorie Chow, David J Norman

**Affiliations:** 1 Department of Plant Pathology, University of Florida, IFAS, Mid-Florida Research and Education Center, 2725 Binion Rd., Apopka, FL 32703, USA; 2 Development Lead North-East Europe, Syngenta Agro GmbH, Am Technologiepark 1-5 63477, Maintal, Germany; 3 Burnham Institute for Medical Research at Lake Nona, 6400 Sanger Road, Orlando, FL 32827, USA; 4 ICBR Proteomics Core, University of Florida, Gainesville, FL 32610, USA

**Keywords:** Bacterial wilt, Temperature, Virulent strains at low temperature, Type VI secretion system, Stress response

## Abstract

**Background:**

*Ralstonia solanacearum*, the causal agent of bacterial wilt, is a genetically diverse bacterial plant pathogen present in tropical and subtropical regions of the world that infects more than 200 plant species, including economically important solanaceous crops. Most strains of *R. solanacearum* are only pathogenic at temperatures between 25 to 30°C with strains that can cause disease below 20°C considered a threat to agriculture in temperate areas. Identifying key molecular factors that distinguish strains virulent at cold temperatures from ones that are not is needed to develop effective management tools for this pathogen. We compared protein profiles of two strains virulent at low temperature and two strains not virulent at low temperature when incubated in the rhizosphere of tomato seedlings at 30 and 18°C using quantitative 2D DIGE gel methods. Spot intensities were quantified and compared, and differentially expressed proteins were sequenced and identified by mass spectrometry (MS/MS).

**Results:**

Four hundred and eighteen (418) differentially expressed protein spots sequenced produced 101 unique proteins. The identified proteins were classified in the Gene Ontology biological processes categories of metabolism, cell processes, stress response, transport, secretion, motility, and virulence. Identified virulence factors included catalase (KatE), exoglucanase A (ChbA), drug efflux pump, and twitching motility porin (PilQ). Other proteins identified included two components of a putative type VI secretion system. We confirmed differential expression of 13 candidate genes using real time PCR techniques. Global regulators HrpB and HrpG also had temperature dependent expression when quantified by real time PCR.

**Conclusions:**

The putative involvement of the identified proteins in virulence at low temperature is discussed. The discovery of a functional type VI secretion system provides a new potential virulence mechanism to explore. The global regulators HrpG and HrpB, and the protein expression profiles identified suggest that virulence at low temperatures can be partially explained by differences in regulation of virulence factors present in all the strains.

## Background

Sudden changes in temperature can induce adaptive shock responses in bacteria, enabling them to colonize widely diverse environments. These adaptive responses involve major changes in the physiological state of the bacterial cells [1,2] allowing them to survive and function in the new environment. Smaller or slower changes in temperature may not induce shock responses but can modulate expression of particular physiological systems such as transport of nutrients, stress responses and virulence efficacy in pathogens. The latter type of change has been studied in animal pathogens that switch expression of colonization and invasion functions when changing hosts [3]. Works addressing temperature-dependent virulence factors in animal pathogens and their regulation have been published only recently [4,5].

Virulence gene expression in most bacteria is modulated by diverse parameters, including pH, ion concentration, growth phase, population density and contact with the host. Temperature may activate or deactivate virulence genes [6], although the mechanisms involved are little known.

Unlike many animal pathogens, plant pathogenic bacteria typically only undergo gradual temperature changes related to seasonal change in environment and are not subjected to sudden changes in temperatures when switching hosts. While most plant pathogens of a given species are active within a limited range of temperature, some strains are virulent across a wider range due to adaptive and perhaps evolutionary pressures. The risk of introducing such strains to new crop zones could be economically devastating. These pathogenicity differences may involve changes in protein regulation or may be due to the presence of unique genes involved in enabling virulence at low temperatures. This is the case of the bacterial wilt pathogen *Ralstonia solanacearum*.

Bacterial wilt is a soil-borne bacterial plant disease which affects more than 200 plant species including solanaceous crops such as tomato and potato [7]. *R. solanacearum*, its causal agent, is a species complex with strains classified into five races based on their host range [8], and into 6 biovars based on biochemical profiles [9]. Strains are further classified into phylotypes and sequevars based on phylogenetic relationships of the internal transcribed spacer region of the 16 s rRNA gene sequence and endoglucanase (egl) gene respectively [10].

A subgroup of *R. solanacearum*, R3B2 Phylotype IIB, includes cold tolerant strains which are able to infect potato at low temperatures [11,12]. This subgroup originated in the highlands of the Andes mountains and is adapted to cooler temperatures than typical strains of *R. solanacearum*[13]. R3B2 strains are designated as “select agent” under the Agriculture Bioterrorism Protection Act of 2002 [14] due to their threat to food security in the U.S. Early reports [13] showed that strains belonging to the R3B2 group could infect potatoes at temperatures as low as 16°C. Recent research has suggested that other races of *R. solanacearum* may also have the ability to survive freezing temperatures and thus have the potential to establish in temperate climates [15,16].

R3B2 are not unique in their ability to infect host plants at low temperatures. We recently reported that R1B1-sequevar 4 strains are also capable of wilting tomato and potato plants at 18°C when soil inoculated [17]. Moreover populations of R1B1 strains are indigenous in tropical and subtropical climates, such as in Florida in contrast to R3B2 strains. A key difference, however, is that while R1B1 sequevar 4 can survive and multiply as well as R3B2 at cool temperatures, they are not as virulent on potato 18°C , although are similarly virulent on tomato. Identifying the factors involved in *R. solanacearum* virulence at low temperatures may provide tools for effective control of the pathogen at low temperatures.

Transcriptome and proteome analysis have been used to identify mRNA and proteins, respectively, present in specific tissues, under diverse conditions. Both methods give a snapshot of the state of the cell at a given time. Because proteins are the key functional molecules, a characterization of the proteome is considered to represent the biological state of a cell more accurately than the mRNA. Current proteomic techniques involve the use of 2D DIGE which can differentiate thousands of proteins by molecular weight and isoelectric point [18]. The use of fluorescent dyes and independent protein controls in 2D DIGE have improved the throughput of 2D gels by allowing multiple proteins to be compared simultaneously. This technique has significantly improved gel-based proteomics analysis, although overall it requires a high quantity (at least 0.6 g) of protein per sample.

In this study we used 2D DIGE gel and MS/MS techniques to compare the protein profiles at 30°C and 18°C of two strains of *R. solanacearum* (UW551 and P673) that are virulent at low temperature with two strains (GMI1000 and P597) that are not virulent at low temperature [17]. We hypothesized that either the difference in regulation of virulence factors present in all the strains or differences due to novel ones present in virulent strains at cool temperatures must explain the difference in virulence at low temperatures. The protein samples were obtained during the colonization phase of the infection from cultures incubated in the rhizosphere of tomato seedlings maintained at 30°C and 18°C. A total of 106 cell-associated and secreted proteins differentially expressed in one or more strains were identified, annotated and discussed.

## Results

### Comparative protein profiles of *Ralstonia solanacearum* strains at 30°C and 18°C

Comparison at 30°C and 18°C of cell-associated proteins from strain P597 incubated in rich media in absence of tomato plants identified 61 protein spots differentially expressed in a preliminary experiment. Fifteen (15) differentially expressed spots with abundant volume were selected for sequencing, and 9 proteins were identified (Additional file 1). The list includes several proteins related to metabolism and transport, a porin part of the type IV twitching motility apparatus (PilQ), and a catalase (KatE), which have been previously related to virulence of *Ralstonia solanacearum*.

Cell-associated comparative protein profiles at two temperatures obtained from bacterial cultures of strains GMI1000, P597, P673 and UW551 incubated in contact with tomato seedlings rhizosphere revealed 872 differentially expressed protein spots, determined by statistically differential intensities for each strains at two temperatures. Further, secreted comparative protein profiles extracted from cultures of strains GMI1000 and UW551 in contact with tomato seedlings rhizosphere identified 172 spots differentially expressed. Overall, a total of 1044 differentially expressed protein spots for all the rhizosphere colonization experiments were identified. Out of the 1044 spots, 418 were selected for protein sequencing. The selection criteria are described in detail in Methods.

A total of 101 unique proteins were identified (Additional file 2). Detailed information about the proteins identified, including number of peptides, protein coverage, statistical confidence, size and isoelectric point is presented by strain in Additional file 3.

**Figure 1:**
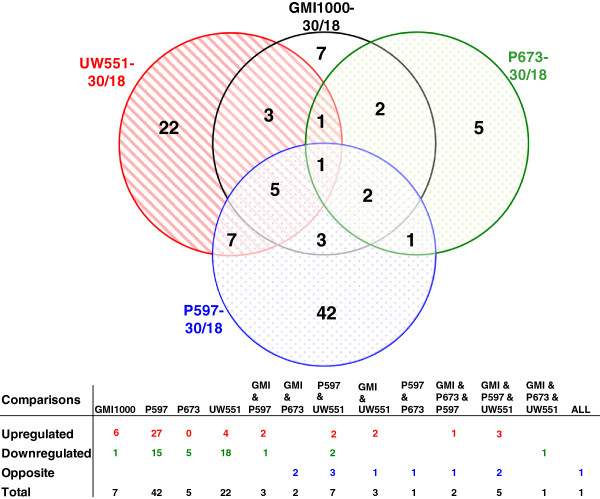
**Venn diagram showing the number and relationship of proteins differentially expressed for the temperature comparisons.** Circles represent the set of proteins differentially expressed for the comparison in the label. The number of proteins differentially expressed is indicated in each set or subset. Tables underneath the comparisons indicate the regulation of the differentially expressed proteins.

The distribution of the identified proteins by regulation shows that (Figure 1) 76 out of the 101 proteins were expressed differentially in individual strains. For example, 22 proteins were differentially produced at 30°C compared with 18°C only in UW551. The selection of the spots was focused on proteins that are down-regulated at 18°C in strains not virulent at low temperature (P597, GMI1000) and/or up-regulated in strains virulent at low temperatures (P673, UW551). In P597 and GMI1000 ≥ 67% of the differentially expressed proteins are more abundant at 30°C. In contrast, 85% of the proteins are more abundant at 18°C in the strains P673 and UW551.

### Functional distribution

**Figure 2:**
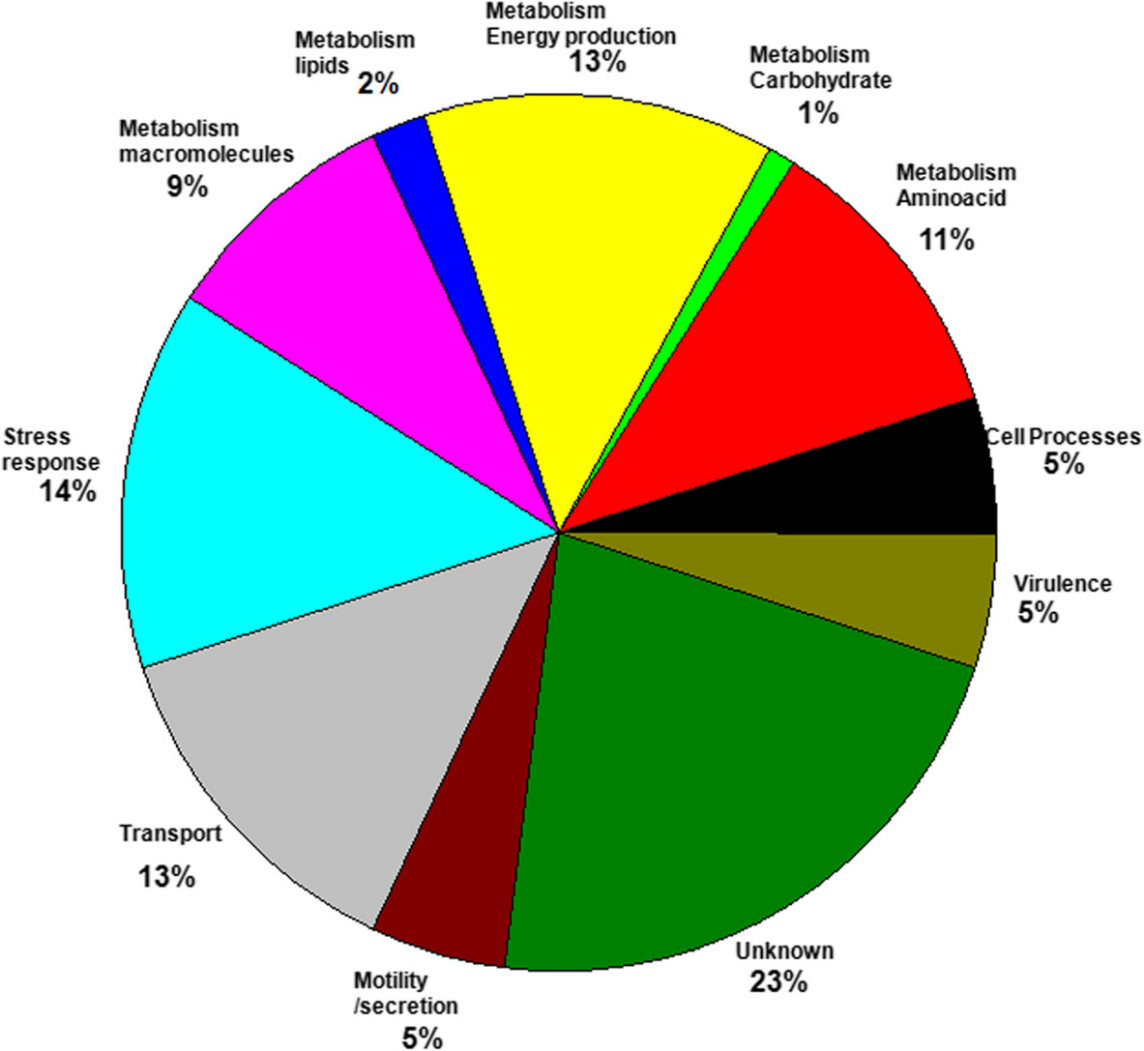
**Distribution of all sequenced differentially expressed proteins by biological process categories.** Pie diagram indicates the percentage of proteins identified in the labeled category. Categories are defined per amiGO database in the Gene Ontology web page (http://www.geneontology.org/).

Identified proteins were annotated, classified, and listed by broad biological categories using the amiGO annotation database [19,20] (Additional file 2). The unknown proteins represent 23% of the identified proteins, and the most abundant (Figure 2). This group includes proteins with only predicted domains that indicate general enzymatic functions, or inferred protein structure, such as transmembrane proteins; however, no specific biological process functions were assigned. Overall, 41% of the identified proteins belong to the metabolism and cell processes categories (Figure 2). The abundance of metabolism related proteins indicates temperature dependent responses in addition to common responses to limited nutritional compounds and exposure to an oxidizing environment from plant exudates [21]. In this category, amino acid, energy production, and macromolecules subcategories are the most numerous. The stress responses category includes proteins with detoxification functions such as catalases, peroxidases, drug or multicopper efflux pumps, chaperones involved in protein production and folding, and putative phasins involved in utilization of polymer reserve granules. Transport category includes movement of inorganic molecules, such as phosphate, transport of amino acids, and sugar derivatives, probably linked to acquisition of nutrients in stressful environments. The smaller categories in terms of numbers although containing important proteins for virulence, were motility, secretion, and virulence.

The classification of proteins by comparative abundance profile at 30°C and 18°C (Table 1) helped to identify patterns of regulation. Of interest in the search for candidate virulence factors at low temperature are groups 2, 4 and 8 (Table 1; Additional file 2) which combine proteins more abundant at 30°C in strains not virulent at low temperatures and/or more abundant at 18°C in strains virulent at low temperatures.

**Table 1:** Temperature dependent proteins grouping criteria

**Group number**	**Grouping criteria**
1	Proteins that are more abundant at 30°C in all type of strains
2	Proteins that are more abundant at 30°C in at least a strain that **is not** virulent at low temperature
3	Proteins that are more abundant at 30°C in at least one strain that **is** virulent at low temperature
4	Proteins that are more abundant at 30°C in at least a strain that **is not** virulent at low temperature and are more abundant at 18°C in at least one strain that **is** virulent at low temperature
5	Proteins that are more abundant at 30°C in at least a strain that **is** virulent at low temperature and are more abundant at 18°C in at least one strain that **is not** virulent at low temperature
6	Proteins that are more abundant at 18°C in all type of strains
7	Proteins that are more abundant at 18°C in at least one strain that **is not** virulent at low temperature
8	Proteins that are more abundant at 18°C in at least one strain that **is** virulent at low temperature
9	No pattern

Criteria used to group proteins by their temperature regulation based on the virulence of strains at low temperature.

We selected a core of 22 proteins that belong to groups 2, 4, 8, and/or have been related to virulence in previous works (Table 2). These proteins are strong candidates as factors that may affect virulence of *R. solanacearum* at low temperatures directly or indirectly.

**Table 2:** A selected subset of candidate virulence proteins whose expression is temperature dependent

**Biological process category**	**Protein name**	**Putative function**	**Protein accession GMI1000**	**Gene tag in GMI1000**	**Profile of regulation group**
**Metabolism**	**Mdh**	Malate dehydrogenase. Glyoxylate cycle	17546717	Rsc1998	4
	**LeuC**	Isopropylmalate isomerase large subunit. Amino acid biosynthesis leucine pathway	17546709	Rsc1990	4
**Stress response**	**ClpB**	ATP-dependent protease. ClpB are chaperones to proteins tagged for destruction.	17546054	Rsc1335	2
	**PpO**	Polyphenol Oxidase. Contributes to resistance to phenolic compounds	17545056	Rsc0337	2
	**GroES**	10kDA chaperonin. Folding and assembly of proteins	17545360	Rsc0641	4
	**GroEL**	60kDA subunit chaperonin. Folding and assembly of proteins	17545361	Rsc0642	4
	**PhaP1**	Phasin_2 domain. Usually associates with PHB granules in bacteria.	17546324	Rsc1605	4
	**HtpG**	Putative heat-shock 90. Molecular chaperone.	17545709	Rsc0990	2
	**Dps**	DNA protection starvation protein.	17547406	Rsc2687	2
	**KatE/katB**	Catalase I hydroperoxidase HpiI oxidoreductase.	17549800	Rsp1581	1
	**KatG**	Heme-dependent peroxidase.	17545494-495	Rsc0775-0776	4
**Transport/motility/secretion**	**SecB**	Putative translocase. Sec dependent pathway	17545075	Rsc0356	4
	**PilQ**	Porin, part of type IV twitching motility system	17547690	Rsc2971	2
	**Rsp0744**	Associated with bacterial type VI secretion apparatus secretion	17548965	Rsp0744	2
	**Hcp**	Type VI secretion system translocator, Hcp1 family	17548966	Rsp0745	4
**Unknown**	**Rsc1727**	Hypothetical protein with an EmrB/QacA family multidrug resistance transmembrane protein	17546446	Rsc1727	2
	**Rsp0167**	Homolog to RaxST PAMP effector in *Xanthomonas oryzae* pv. *oryzae*.	17548388	Rsp0167	2
**Virulence**	**Egl**	Endoglucanase. Degradation of polysaccharides	17548383	Rsp0162	3
	**Rsp0275**	Putative chitinase	17548496	Rsp0275	1
	**ChbA**	Putative exoglucanase. Cellobiohydrolase A domain	17548804	Rsp0583	2
	**HrcC**	Secretin of the conserved family of type III apparatus system	17549095	Rsp0874	8
	**WecC(EpsD)**	Enzyme part of the exopolysaccharide operon (EpsD).	17549237	Rsp1016	8

### mRNA relative expression of 13 candidate cool virulence factors and global regulators *hrpG* and *hrpB* correlate with protein expression profiles

**Figure 3:**
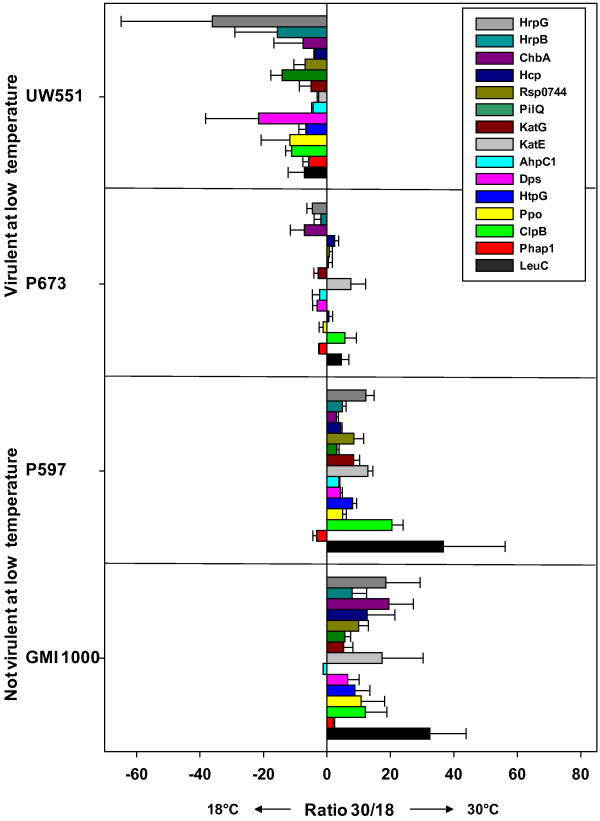
**Relative expression of 15 genes for GMI1000, P673, P597, and UW551 at two temperatures.** Positive bars represent higher expression at 30°C and negative bars represent higher expression at 18°C. Quantitative reverse transcription-polymerase chain reaction was performed on equal amounts of total RNA per sample extracted from bacterial suspensions grown in co-culture with *in vitro* tomato seedlings. 16sRNA was used as reference for the relative expression of each gene. Bars represent standard error of three biological replicates.

In order to confirm the proteomics results, the relative expression of 13 genes at 30 and 18°C was quantified by real-time reverse transcription qRT_PCR analysis. The relative expression of each gene to a reference gene (16sRNA) at each temperature was calculated, and presented as the ratio at 30°C versus 18°C (Figure 3). Expression of 12 candidate genes tested was up-regulated at the higher temperature for strain P597. The only gene that was down-regulated at 30°C was *phaP1* while the expected result was up-regulation. In P673, results were variable; however, ratios were low compared with the ratios in the not virulent strains GMI1000 and P597, and most of the genes tested were down-regulated. In UW551 all genes tested were down-regulated at 30°C. The profile in Figure 3 correlates with the expected pattern of expression inferred from the proteomics data for the target genes tested: in P597 and GMI1000 genes are up-regulated at 30°C and in P673 and UW551 genes maintain similar expression at both temperatures or are up-regulated at 18°C, respectively.

Relative expression of the *hrpG* and *hrpB* global virulence regulators was also tested with qRT_PCR (Figure 3). The relative expression at 30°C in P597 and GMI1000 is up-regulated, while in P673 and UW551 it is down-regulated, suggesting a correlation between expression of regulators HrpG and HrpB and virulence at low temperature.

### Functional annotation and comparative temperature profile

In this section proteins differentially expressed and their profiles are described by biological process category.

### Metabolism/cell processes

Cell processes class included proteins related to translation, transcription, and chaperoning (Additional file 2). Most of the proteins in this category belong to groups 2 and 8 (Table 1). Their profile of regulation suggests a more active cell state at 30°C for the strains not virulent at low temperature, and at 18°C for the virulent ones.

In the metabolism category approximately one third of the proteins were related to amino acid biosynthesis. The leucine-isoleucine biosynthetic pathway in particular, was represented by 4 different proteins (LeuC, IlvC, IlvD, ThrC) whose profile of expression belong to groups 2, 4, and 8 (Additional file 2). LeuC is the large unit of the isopropylmalate isomerase, a key enzyme in the L-leucine biosynthesis from valine. The pattern of expression of LeuC was confirmed by qRT_PCR (Table 2, Figure 3). Enzymes that belong to cysteine and glutamate were also more abundant at 30°C in P597.

Energy production is another important subcategory in metabolism. Proteins AceE, GltA, NuoF, and Mqo, related to the TCA cycle have a variable pattern of abundance ratio; however proteins GapA, Mdh, and AceA related to glycolysis belong to groups 2, 4 and 8 (Additional file 2).

Metabolism of macromolecules subcategory represented 9% of the differentially expressed proteins, their abundance profile was variable, and no correlation was observed with virulence at low temperature.

### Stress response

Stress response proteins represented 14% of the differentially expressed proteins (Figure 2). Proteins in this category relate to biosynthesis of carbon and energy reserves, protein folding in stressful environments, and proteins involved in detoxification. Two proteins PhaP2, PhaP1 which contain a phasin domain were differentially expressed. Phasin domains are associated with synthesis or degradation of poly β-Hydroxybutyrate (PHB) granules, formed as an alternate carbon reserve for organisms facing starvation in harsh environmental conditions [22,23]. In *Rhodospirillium rubrum* PhaP protein is an activator of the PHB degradation pathway [24]. In particular, PhaP1 was differentially expressed in all the strains and its abundance profile (Table 2) correlated with virulence at low temperature. The RT-PCR confirmed the pattern of expression of all strains except for P597 where the gene was down-regulated (Figure 3), although with a small ratio.

GroEl/GroEs, form a complex that functions as chaperone in folding and refolding of proteins, usually induced under adverse environmental conditions [25]. Heat-shock protein HtpG and protease ClpB were shown to facilitate *de novo* folding of proteins during high temperature conditions in *E. coli*[26]. Although the temperature changes in *R. solanacearum* are not sudden, these proteins may aid in stabilizing proteins during low temperature conditions (Table 2).

Differentially expressed detoxification proteins whose expression correlate with virulence at low temperature (groups 2, 4, 8) included Ppo, KatG, Dps (Table 2, Additional file 2), Rsc0754, and Rsp1530 (Additional file 2). Ppo is a polyphenol oxidase which has been shown to have strong tyrosinase activity suggesting a possible role in counteracting phenolic compounds produced by the host plants [27]. Dps contributes to oxidative stress tolerance and virulence on tomato plants [28]. KatG and Rsc0754 are predicted peroxidases [21], while Rsp1530, has laccase activity, similar in function to the tyrosinase Ppo [27] and it was up-regulated by hydrogen peroxide in culture and *in planta*[21].

Catalase KatE was more abundant at 30°C in GMI1000, P597 and P673 (Table 2, Figure 3). KatE was also up-regulated at 30°C when strain P597 was grown in rich media (Additional file 1). A homolog of KatE in *Agrobacterium tumefaciens* (KatA) has been shown to contribute to its survival in adverse environmental conditions as in the presence of hydrogen peroxide [29]. In *R. solanacearum*, KatE probably contributes to survival in the presence of phenolic compounds produced by the plant hosts. The response to the oxidative environment is temperature dependent; however, it does not correlate with virulence of the strains at low temperature.

Conversely, AhpC1 predicted peroxidase was more abundant at 18°C in GMI1000, P673 and UW551 (Additional file 2, Figure 3). The ratios of abundance in these comparisons were smaller than for the case of KatE.

### Transport

The pattern of expression of 9 proteins related to transport of inorganic compounds were up-regulated in all the strains compared at 30°C (group 1).Those proteins included putative alkaline phosphatases and several ABC-type transporters (Additional file 2). Their pattern of regulation suggests more ion activity at 30°C irrespective the virulence of the strain at low temperatures. Four proteins related to carbohydrate transport and two related to aminoacid transport were differentially expressed. Their pattern of regulation varied, and there was no obvious co-relation with virulence at low temperatures.

### Motility/secretion

In the subcategory motility only PilQ was identified as differentially expressed (Table 2). PilQ is a porin, integral part of the structural apparatus responsible for the twitching movement of *R. solanacearum*[30]. Twitching motility has been associated with attachment and virulence of *R. solanacearum* on their host [31], and its expression is dependent on the quorum sensing regulatory pathway through PehS/Pehr two components system [31]. PilQ was up-regulated at 30°C in P597 (Additional file 1, Table 2) in rich media and in co-culture with tomato rhizosphere. In the RT_PCR experiment PilQ was up-regulated at 30°C in GMI1000 and P597, and up-regulated or unchanged at 18°C in UW551 and P673 (Figure 3). This profile of expression correlates with virulence at low temperatures. Twitching motility was also temperature dependent, independent of the presence of plants in previous studies [17].

Rsp0744 and Hcp (Rsp0745) were identified as part of a recently described type VI secretion system [32]. Their regulation profile correlates with virulence at low temperature (groups 2, 4) (Additional file 2, Figure 3). Rsp0744 is a homolog to a type VI secretion apparatus protein known as VipB, or VCA0108 in *Vibrio cholerae* , which is thought to be part of the core secretion apparatus [33]. Rsp0745 is a homolog to Hcp of several gram-negative bacteria [34]. This is a protein secreted by the putative type VI secretion system [32,33] and thought to be a translocator [32,35].

Type VI secretion systems have only recently been described in animal and plant pathogens [32,33,36,37]. This system is linked to virulence in *Vibrio cholerae* and *Pseudomonas aeruginosa*. Since then the type VI secretion system has also been identified *in silico* in many gram negative bacterial genomes including *R. solanacearum*[34]. Similarly to other secretion systems it is also widespread among gram-negative bacteria [38]. Interestingly, a cluster called *imp* with homology to the later described type VI secretion was shown to have a role in temperature-dependent protein secretion in *R. leguminosarum*[39], congruent with our results. The presence and pattern of abundance of these proteins, plus the involvement in virulence of type VI secretion systems in other bacterial pathogens, make the identified type VI secretion a strong candidate to virulence factor at low temperatures in *R. solanacearum*.

### Unknowns

Among the twenty three unknown proteins that were differentially expressed, a cell-associated hypothetical Rsp0167 (Table 2) was considered of interest because is a homolog of raxST, a component of a type I secretion cassette AvrXa21 in *Xanthomonas oryzae* pv. *oryzae*[40]. In this latter organism the cassette contains four genes: *raxA*, *raxB*, *raxC* and *raxST*, which encode a sulfotransferase-like protein and are necessary for recognition of Xa21, a receptor-like kinase in rice. This putative pathogen-associated molecular pattern (PAMP) effector could be involved in virulence in *R. solanacearum*. Rsp0167 was down-regulated at 18°C in GMI1000.

Another protein of interest is Rsc1727 (Table 2). This is a hypothetical protein with an EmrB/QacA family multidrug resistance domain. This subfamily of drug efflux proteins are related to drug/antibiotic resistance transporters. Transporters members of this subfamily include multidrug resistance locus in *Escherichia coli* and *Vibrio cholerae*[41,42].

Although the experimental design favored identification of proteins that are present in all the strains, two differentially expressed proteins absent in GMI1000 were identified: RRSL_00447 and RSPO_m01224 (Additional file 2). The first has a methyltransferase domain and is present in UW551, P597 and P673. It was up-regulated at 30°C in P597. The second is annotated as a hypothetical protein and is present in P597, P673, and in sequenced *R. solanacearum* strain Po82 [43]. It was up-regulated at 18°C in P673.

### Virulence

Secreted virulence factors identified included a putative cellobiohydrolase encoded by the *chbA* gene, an endoglucanase (Egl) and a putative chitinase (Rsp0275) (Table 2). Their function is related to attack and degradation of the host’s cellular walls. It has been suggested that ChbA attacks the hemicellulose fraction of plant cell walls [44]; however, *in vitro* enzyme activity on regular exoglucanase substrates such as methyl-umbelliferyl-cellobioside (MUC) has never been demonstrated [44]. Liu *et al.*[45] demonstrated that a GMI1000 strain defective in ChbA production is less virulent when soil inoculated. In the same way, an Egl mutant impaired in endoglucanase activity is also less virulent [45]. Their data suggest that ChbA and Egl contribute equally to virulence in an additive fashion [45]. It was expected that ChbA and Egl would be co-regulated. The proteomics and RT_Pcr results do not support this assumption. In our results, ChbA was more abundant at 30°C in GMI1000, and Egl was more abundant at 30°C in UW551 (Additional file 2); however, ChbA was more abundant at 18°C in UW551 in the RT_PCR results (Figure 3).

The cell-associated proteins HrcC and WesC were more abundant at 18°C in UW551 (group 8 in Table 2), supporting a role in virulence at low temperature. HrcC is an integral part of the type III secretion apparatus which is a determinant virulence factor in *R. solanacearum*[46], and WesC (EpsD) is part of the exopolysaccharide operon, involved in aggressiveness during host colonization [47].

## Discussion

In previous work we determined that there is a barrier to virulence at low temperature at the stage of colonization of the host rhizosphere [17]. Therefore in this study we focused our comparative analysis on that stage of the disease. We designed an *in vitro* system that mimics the natural rhizosphere conditions and at the same time facilitate the retrieval of *R. solanacearum* proteins from a sterile environment. Bacteria in co-culture with tomato seedlings exploit plant exudates in order to multiply and approach roots as in the natural rhizosphere [48,49]. We used 2D-DIGE gels to identify proteins that were differentially expressed in 4 strains of *R. solanacearum* that are either virulent or not virulent at low temperatures. Our experiments provided information about the identity of proteins expressed differentially and their pattern of regulation at two temperatures. Following we discuss the biological significance of our data.

Overall, the abundance of proteins related to cell processes and metabolism at low temperature suggests that the metabolic state of virulent strains is not reduced at low temperature as it is for not virulent strains. For example, the leucine-isoleucine biosynthetic pathway could be important for survival, growth, or competition of *R. solanacearum* in the rhizosphere environment, and strain UW551 which increases its expression at 18°C may have a competitive advantage for growth and colonization of the roots at low temperature over the other strains. The glycolysis process may also contribute to fitness of virulent strains at low temperature in the rhizosphere.

The presence of stress response proteins was expected because *R. solanacearum* encounters an oxidative environment in the rhizosphere of the host plant [21]; however, these proteins were also temperature dependent and the strains responded differently to this environment at low temperatures. The different behaviors between KatE, AhpC1, and other predicted peroxidases suggest that the different catalases/peroxidases may have specific functions and may be activated/de-activated by different regulators. The higher volumes of heat-shock protein HtpG and protease ClpB in strains that are virulent at low temperatures, suggest a higher level of cellular protection at low temperatures in virulent strains.

The presence of an Hcp homolog in our results among the secreted proteins indicated a functional type VI secretion system. Secretion of Hcp is used to identify functional type VI secretion systems in bacterial species [35,50] since presence of Hcp in the secretome of bacterial species is required for the function of this secretion system [35]. An important observation is that the regulation of this system is temperature dependent similar to the *imp* cluster in *Rhizobium leguminosarum*[39]. The pattern of expression of the type VI related proteins suggests that this system may play a role in virulence at low temperature.

The difference in pattern of regulation of the virulence proteins identified suggests different regulation pathways. This was partially determined for ChbA, Egl, Eps and Tek [51]. Egl and Tek are directly regulated by the global regulator PhcA [52], while ChbA, and Eps are regulated indirectly through a VsrA/VsrD two component system [53].

Global virulence regulators HrpG and HrpB expression was found to be temperature dependent and correlated with virulence at low temperature (Figure 3). Our results support the hypothesis that virulence at low temperatures is explained primarily by differences in temperature dependent regulation of proteins present in all the strains. *R. solanacearum* has a complicated regulation network that it is still largely unknown. There may yet be regulation subsystems that control specific virulence and survival factors at low temperatures. The future direction is to investigate the known global virulence regulators and perhaps identify new ones.

Proteomics is a powerful method to give a picture of the state of an organism and discover proteins that may contribute to specific functions; however the presence of very abundant proteins obscured the identification of proteins produced in smaller quantities. For example, we noted the absence of type III effectors in our results. We identified a putative porin HrcC associated with the type III apparatus, which suggest that type III effectors cannot be discarded as potential temperature dependent proteins. An indication that the type III secretion may be temperature dependent was the profile of the type III global regulator HrpB in our qRT_PCR expression experiment.

We sequenced 40% of the differentially expressed spots, reducing our capacity to identify all the protein spots that were differentially expressed in the comparisons. We hypothesized that a protein important for virulence should be expressed in less quantity at low temperature for not virulent strains, and in more quantity for virulent ones at low temperatures; therefore most protein spots that fit the criteria were selected for sequencing. We combined experiments to obtain a more complete picture with very good results. Very few of the proteins were contradictory across experiments and the rest were independent or congruent, providing us with an extra quality control step.

## Conclusions

The comparative protein analysis presented in this work identified likely candidates for temperature dependent virulence factors. To our knowledge, this is the first report of temperature dependent protein expression in a plant pathogen. This also is the first report of an inferred functional system in *Ralstonia solanacearum*. This secretion system was previously identified by bioinformatic methods but not previously confirmed. Our results support the hypothesis that virulence at low temperature is mainly due to regulation differences between strains that are virulent and strains that are not virulent at low temperatures. This work provides a list of candidate virulence factors at low temperatures whose expression is temperature dependent and a working hypothesis for the study of the virulence mechanism at low temperatures.

## Methods

### Experimental design

**Figure 4:**
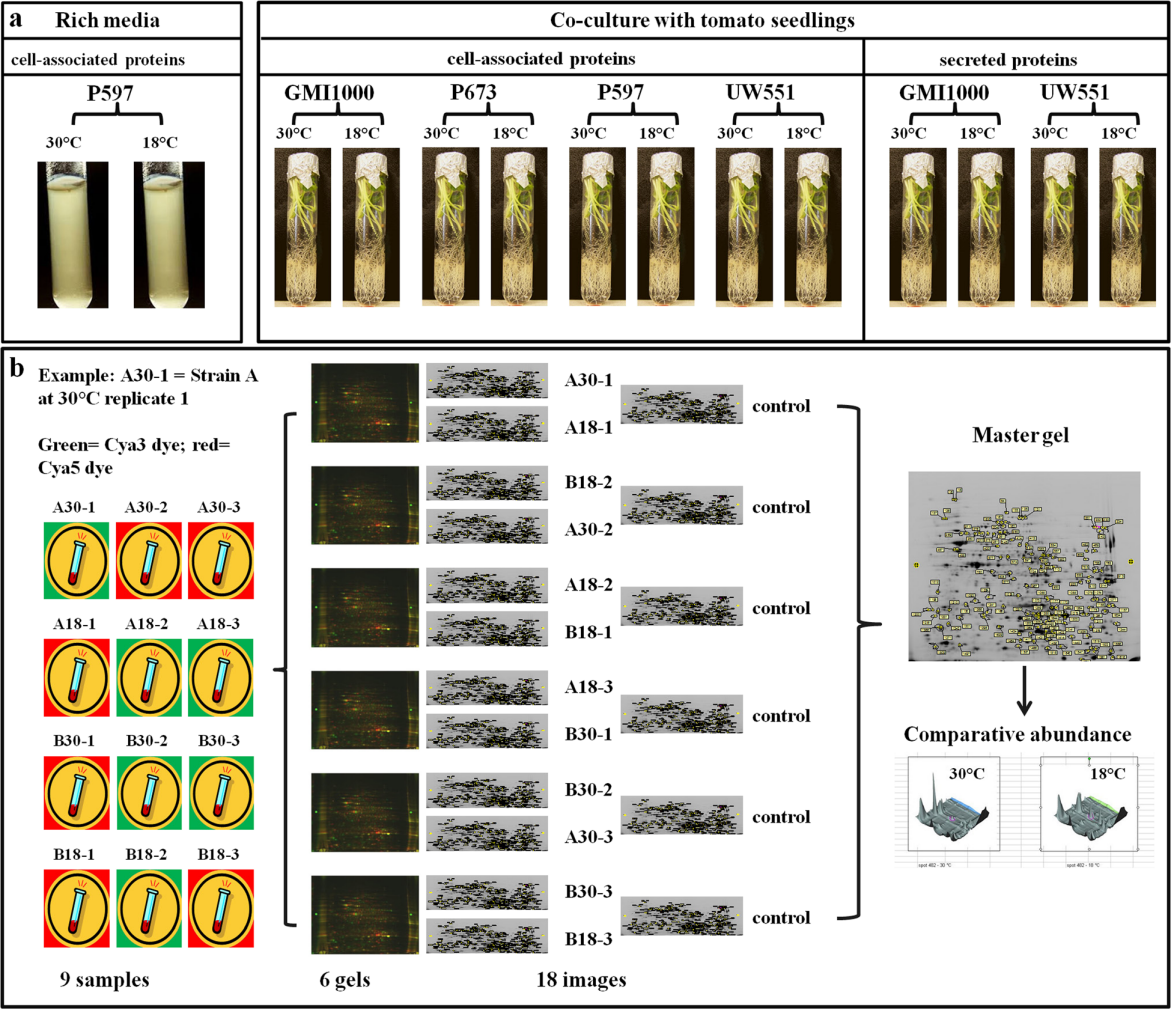
**Experimental design flowchart. a**. Conditions of bacterial populations and type of proteins extracted by comparisons. **b**. Example of experimental design for the comparison of two strains. Protein samples to be compared were stained with either Cy3 or Cy5 fluorescent dyes. Three biological reps of each sample to be compared were combined in several gels to provide statistical power for comparisons. The pool of gels is normalized in order to compare spots from different gels. Spots were identified and localized across gels and their abundance compared statistically. A list of differentially expressed spots was analyzed and a subset of the total number of spots of interest were excised and sequenced by MS analysis.

We compared cell-associated proteins in separate experiments: 1) P597(R1B1, Sequevar 18), obtained from bacterial populations grown *in vitro* in 20 ml of rich media at 30°C and 18°C without plants in one experiment. 2) UW551 (R3B2, Sequevar 1), P673 (R1B1, Sequevar 4), GMI1000 (R1B3, Sequevar 38), and P597, obtained from bacterial populations incubated in contact with the rhizosphere of sterile tomato seedlings (see details in next section), in three experiments with different combinations of strains (Figure 4a).

We also compared secreted proteins of UW551 and GMI1000 extracted from the liquid medium when bacterial populations were incubated in contact with the rhizosphere of sterile tomato seedlings in one experiment (Figure 4a).

Each comparative experiment included three biological replicates per treatment per strain (30°C and 18°C) that were loaded in ***n*** comparative gels, where ***n*** is the number of strains compared in each experiment by the number of biological replicates. Each comparative gel was loaded with two samples and an internal control (see 2D DIGE protein gels section) in different combinations. Figure 4b exemplifies the experimental design for the comparison of two strains: we started with 6 samples at 30°C and 6 at 18°C. The samples were loaded in 6 gels in different combinations dyed with Cya3 and Cya5, including comparisons of the same strain at two temperatures and different strains in the same gel. The 6 gels produced 18 images including the internal controls and samples (Figure 4b). All images were matched and normalized. A master gel was created with information from all gels. Temperature protein profiles were compared using t-tests.

A subset of approx. 40% of the differentially expressed proteins was selected for MS/MS analysis. The following criteria were used for the selection: 1- Sufficient protein spot volume for sequencing; 2- More abundant at 30°C than at 18°C for the strains not virulent at 18°C (P597 and GMI1000); 3- More abundant at 18°C than at 30°C for the strains virulent at 18°C (P673, UW551).

Since one spot could potentially represent multiple proteins, the best fit protein for each spot was determined by selecting the best percentage of confidence (≥99%) for the peptide assignation and best coverage of the protein using the protein software Scaffold (Proteome Software Inc., Portland, OR). Proteins with conflicting results were eliminated.

### *In vitro* tomato seedlings and inoculation with *R. solanacearum*

Tomato cultivar Walter (*Solanum lycopersicon* ‘Walter’) plants were grown as previously described [17]. Seeds were germinated on water agar plates (Bacto Agar at 16.0 g/liter, pH 7.0). Seedlings were transferred to test tubes containing 20 ml of MSMO liquid medium (Murashige-Skoog plus organics, Sigma M6899) supplemented with sucrose at 30 g/liter, pH 5.8. Plants were grown *in vitro* about 10 weeks. To establish bacteria-plant root co-cultures *in vitro*, *R. solanacearum* populations were cultured in casamino acids-peptone-glucose (CPG) liquid medium (5.0 g/liter glucose, 1.0 g/liter casamino acids, 10.0 g/liter peptone) to an exponential phase (OD600 = 0.8). Each test tube containing five tomato plantlets was inoculated with 200 μl of the cell suspension for a final concentration of approx. 8 × 10^6^ cells/ml. Co-cultures were incubated for 5 days at 18°C and for 2 days at 30°C, both on a rotary shaker (150 rpm).

### Bacterial sample collection, protein extraction, and CyDye labeling

Bacterial suspension in MSMO media was collected from the *in vitro* tomato seedlings tubes in sterile flasks, and then filtered with a 20 μm Steriflip filter (SCNY00020 Millipore Corp.) to eliminate plant debris. The resulting suspension was centrifuged for 10 minutes at 8000×g to collect the bacterial pellet. This pellet was resuspended in 500 μl of DIGE buffer (8 M urea, 2 M thiourea, 4% CHAPS, 0.2% SDS, and 10 mM Tris HCl pH 8.5), fast frozen and stored at −80°C for further processing. The supernatant was filtered again with a 0.22 um Steriflip filter (SEIM179M6 Millipore Corp.) to eliminate any residual bacteria and concentrated at 4°C with Amicon Ultra-15 30 K (Millipore UCF903024) filter. Concentrated bacterial secreted protein was extracted and cleaned from salts and other contaminants with acid phenol according to Nissinen et al. [54]. The contaminant-free protein pellet was dissolved in 250 μl of DIGE buffer. Protein sample solutions were further clarified by ultracentrifugation at 40 k × g at 15°C for 30 min. Protein concentrations were measured with the EZQ Protein Assay kit (Invitrogen) after adjusting the protein solution to pH 8.5.

To label the protein samples, 1 μl of CyDye (400pmole) diluted in DMF was added to 100 μg of protein and incubated on ice in the dark for exactly 30 min. To stop the reaction, 1 μl of 10 mM lysine was added and incubated for 10 more minutes.

### 2D DIGE protein gels

For each gel, 100 μg of Cy2 labeled internal reference, 100 μg of Cy3 labeled control sample, and 100 μg of Cy5 labeled experimental sample were mixed before rehydration of IPG strip (GE healthcare). Dye swapping was used and sample mixtures for the gels were frozen at −80°C until ready for 2D GE.

Before IPG strip rehydration, DTT concentration of each sample mixture was adjusted to 100 mM, IPG buffer pH 3 to 11 to 0.5%, and final volume to 500 μl with labeling buffer. A small amount of Orange G was also added as tracking dye. A non linear IPG strip (24 cm pH 3 to 11) was rehydrated O/N with 500 μl of IEF sample mixture in the dark at room temperature.

First Dimension IEF run was carried out in an IPGphor 3 unit (GE Healthcare) under a layer of mineral oil with gel surface at 19°C in the dark. Electrodes were placed on each end over pieces of filter paper dampened with Milli Q water. The initial voltage was set at 500 V for 1500 Vhr (voltage × hour). Voltage then was ramped up to 1000 V in 6000 Vhr, ramped up to 8000 V in 13.5 kVhr, then ramped up to 10000 V in 16.5 Vhr, and finally was focused at 10000 V for 80 kVhr until it reached a steady state of around 27 μA.

After completion of IEF, proteins in each strip were first reduced in 15 ml of a solution composed of 50 mM Tris–HCl pH 6.8, 6 M Urea, 30% (v/v) glycerol, 2% (w/v) SDS, and 100 mM DTT , for 20 min in the dark at room temperature, then were alkylated in 15 ml of 50 mM Tris–HCl pH 6.8, 6 M Urea, 30% (v/v) glycerol, 2% SDS, and 2.5% idoacetamide for 20 min at room temperature in the dark. After reduction and alkylation, the strip was transferred and was mounted on a 8 to 16% precast Tris Glycine polyacrylamide gel which was cast between two low fluorescent glass plates under a layer of warm 0.5% agarose made in SDS electrophoresis running buffer. Electrophoresis was first run at 12°C and 10 mA/gel for one hour, and then O/N at 12 mA/gel with a limit of 150 V until the dye front reached the bottom of the plate.

Immediately after gel electrophoresis, gel cassettes were rinsed with deionized water and dried with lint-free toweling before being scanned with a Typhoon 9400 Variable Mode Imager (GE Healthcare). The excitation/emission wavelengths for Cy2, Cy3 and Cy5 were 488/520, 532/580 and 633/670 nm respectively. Three images (internal reference, control and experimental) were acquired for each gel. Examples of gels and images for a comparison of P597 and GMI1000 cell-associated proteins are presented in Additional file 4 to illustrate the process.

The digital image information acquired was then analyzed with DeCyder 2D version 7.0, automated image analysis software by GE Healthcare. All spots presented in all images of all gels were co-detected, matched and normalized with DIA Module (Differential In-Gel Analysis). Information from replicate gels was analyzed with BVA Module (Biological Variation Analysis). In BVA, a master gel was created with information from all gels, with matches of multiple images from different gels to provide statistical data for differential protein expression levels between control and experimental groups. Protein spots of interest (POI) were selected by setting the fold difference threshold to 1.5 folds. Any protein spot from the experimental group (18°C) expressed under or over 1.5 folds when compared with spots from the control group (30°C) was selected as POI. A protein spot pick list was made after filtering the spot information based on matching quality, appearance in all gels, and statistical confidence when Student’s T-test *p* value is equal to or less than 0.05. Excision and Digestion of protein spots was done as follows: with a set of paper reference circles attached to each side of the glass plate, the ordinance information for each POI was translated and transferred to an automatic spot picker (ProPic by Genomic Solutions) through the pick list. Spots then were excised by the picker and transferred to a 96-well collecting plate. Protein spots were washed and destained, proteins in the spot were first reduced by DTT and alkylated by 40 mM Iodoacetamide before O/N trypsin digestion at 37°C.

### MS/MS sequencing

The enzymatically digested samples were injected onto a capillary trap (LC Packings PepMap) and desalted for 5 min at a 3 μl/min flow rate of 0.1% v/v acetic acid. The samples were loaded onto an LC Packing® C18 Pep Map nanoflow HPLC column. The elution gradient of the HPLC column started at 3% solvent B and 97% solvent A, and finished at 60% solvent A and 40% solvent B for 30 min for protein identification. Solvent A consisted of 0.1% v/v acetic acid, 3% v/v ACN, and 96.9% v/v H_2_O. Solvent B consisted of 0.1% v/v acetic acid, 96.9% v/v ACN, and 3% v/v H_2_O. LC-MS/MS analysis was carried out on a hybrid quadrupole-TOF mass spectrometer (QSTAR elite, Applied Biosystems, Framingham, MA). The focusing potential and ion spray voltage was set to 225 V and 2400 V, respectively. The information-dependent acquisition (IDA) mode of operation was employed in which a survey scan from m/z 400–1800 was acquired followed by collision induced dissociation (CID) of the four most intense ions. Survey and MS/MS spectra for each IDA cycle were accumulated for 1 and 3 s, respectively.

For the protein search algorithm, tandem mass spectra were extracted by ABI Analyst version 2.0. All MS/MS samples were analyzed using Mascot (Matrix Science, London, UK; version 2.2.2). Mascot was set up to search NCBI with taxonomy Bacteria database assuming the digestion enzyme trypsin. Mascot was searched with a fragment ion mass tolerance of 0.50 Da and a parent ion tolerance of 0.50 Da. Iodoacetamide derivatives of Cys, deamidation of Asn and Gln, oxidation of Met, are specified in Mascot as variable modifications. Scaffold (Proteome Software Inc., Portland, OR) was used to validate MS/MS based peptide and protein identifications. Peptide identifications were accepted if they could be established at greater than 95.0% probability as specified by the Peptide Prophet algorithm [55]. Protein identifications were accepted if they could be established at greater than 99.0% probability and contained at least 3 identified unique peptides. Protein probabilities were assigned by the Protein Prophet algorithm [56].

### Total RNA extraction and cDNA synthesis

Cell samples from bacterial co-cultures with tomato roots were extracted as follows: the suspension was filtered with a 20 μm Steriflip filter (Millipore Corp.) to eliminate all plant debris. Filtrate was centrifuged at 8000 × g and pellet was collected and suspended in RNA Bacteria Protect Reagent (Qiagen Cat. 76506) following the manufacturer’s instructions. Prepared samples were stored at −70°C for further processing. Total RNA was extracted from the stored samples as per Jahn et al. [57]. Quantity of extracted RNA was measured using the Nanodrop 2000C spectrophotometer. Quality was assessed on denaturing electrophoresis gels. Purified RNA was reverse-transcribed to cDNA using the SuperScript® Vilo^TM^ cDNA Synthesis Kit (LifeTechnologies Cat. 11754–050), according to manufacturer’s instructions. Quality and quantity of cDNA were assessed by spectrophotometry (Nanodrop Technologies, Inc.).

### Real time qRT-PCR relative quantification and primer design

Primers were designed for a 188-bp fragment of the 16sRNA sequence and fragments ranging from 100 bp to 250 bp of the target genes (Additional file 5). Preliminary experiments were made with three dilutions of each cDNA samples to find the best dilution for the relative quantification. Relative qRT-PCR was performed using the Roche Lightcycler 480 with the SYBR Select Master Mix Kit (Life Technologies Cat. 4472908) according to the manufacturer’s instructions. Samples of 250 ng were used for the amplification using the program: for primers with Tm > =60°C (UDG activation 50°C for 2 min, Amplitaq activation 95°C for 2 min, and 40 cycles of denaturation at 95°C for 15 sec and anneal/extend at 60°C for 1 min). Melting curves of the samples were assessed to evaluate contamination. The target gene/16sRNA ratio of amplification at 18°C was normalized against the ratio of each strain at 30°C for the relative quantification analysis.

### Availability of supporting data

The data sets supporting the results of this article are included within the article and its additional files. The protein and peptide data sets supporting the results are presented in Additional file 6.

## Abbreviations

R3B2: Race 3 Biovar 2; R1B1: Race 1 Biovar 1; 2D-DIGE: Two-dimensional differential gel electrophoresis; MS/MS: Tandem mass spectrometry; PCR: Polymerase chain reaction; qRT_PCR: Quantitative reverse transcription polymerase chain reaction; mRNA: Messenger ribonucleic acid; PHB: Poly β-Hydroxybutyrate; TCA: Tricarboxylic acid cycle; MUC: Methyl-umbelliferyl-cellobioside; CHAPS: 3-[(3-cholamidopropyl) dimethylammonio]-1-propanesulfonate; IPG: Immobilized pH gradient; DTT: Dithiothreitol; IEF: Isoelectric focusing; SDS: Sodium dodecyl sulphate.

## Competing interests

The authors declare that they have no competing interests.

## Authors’ contributions

AMB designed the experiments, performed the inoculations, bacterial and secreted proteins preliminary extraction, primer design, RNA extraction, qRT-PCR experiments, data analysis and interpretation, and wrote the manuscript. UCMA and AMN conceived the co-culture in tomato method, designed and performed the preliminary experiments. MC performed the 2D-DIGE protein comparisons and determination of the spots differentially expressed. DJN conceived of the study, participated in all steps of the project as coordinator and critically reviewed the manuscript. All authors read and approved the final manuscript.

## Supplementary Material

Additional file 1 **Proteins produced differentially at 30 and 18°C in strain P597 when incubated in absence of plants.** List of proteins produced differentially at 30 and 18°C in strain P597 of *R. solanacearum* when incubated in rich media, preliminary experiment. The information for each protein includes name, putative function, broad biological process category, equivalent protein accession number and gene tag in GMI1000 and UW551, predicted cellular localization, and regulation. Click here for file

Additional file 2 **Proteins produced differentially at 30 and 18°C in strains GM1000, P597, P673, and UW551 of *R. solanacearum *in co-culture with tomato seedlings.** List of proteins produced differentially at 30 and 18°C in strains GM1000, P597, P673, and UW551 of *R. solanacearum* when incubated in contact with *in vitro* tomato plants roots. The information for each protein includes name, putative function, broad biological process category, equivalent protein accession number and gene tag in GMI1000 and UW551, predicted cellular localization, and regulation. Click here for file

Additional file 3 **Best fit protein detailed identification information by strain.** The information for each spot sequenced includes regulation at 30°C, ratio of expression compared with 18°C, isoelectric point, from the gels; best fit protein description, accession number, protein size in Da, number of peptides, and protein coverage from Scaffold. Click here for file

Additional file 4 **Gel images for comparative experiment of two strains to illustrate the process of gel comparisons.** Includes comparative gels and images with spots numbered and normalized for GMI1000 and P673 comparative gels of cell-associated proteins. Click here for file

Additional file 5 **List of primers designed for the qRT-PCR relative quantification.** Includes the primer pair sequences used for qRT-PCR and the estimated length of the products. Click here for file

Additional file 6 **Peptide report from Scaffold.** Excel workbook containing 5 worksheets with the complete peptide report for the proteins by experiment exported from Scaffold viewer program. Click here for file

## References

[B1] Hurme R Rhen M Temperature sensing in bacterial gene regulation - what it all boils down to Mol Microbiol 1998 15 1 1 6 10.1046/j.1365-2958.1998.01049.x 9786180

[B2] Ramos JL Gallegos MT Marques S Ramos-Gonzalez MI Espinosa-Urgel M Segura A Responses of gram-negative bacteria to certain environmental stressors Curr Opin Microbiol 2001 15 2 166 171 10.1016/S1369-5274(00)00183-1 11282472

[B3] Konkel ME Tilly K Temperature-regulated expression of bacterial virulence genes Microbes Infect 2000 15 2 157 166 10.1016/S1286-4579(00)00272-0 10742688

[B4] Rejasse A Gilois N Barbosa I Huillet E Bevilacqua C Tran S Ramarao N Arnesen LPS Sanchis V Temperature-dependent production of various PlcR-controlled virulence factors in *Bacillus weihenstephanensis* strain KBAB4 Appl Environ Microbiol 2012 15 8 2553 2561 10.1128/AEM.07446-11 22307282 PMC3318839

[B5] Kimes NE Grim CJ Johnson WR Hasan NA Tall BD Kothary MH Kiss H Munk AC Tapia R Green L Detter C Bruce DC Brettin TS Colwell RR Morris PJ Temperature regulation of virulence factors in the pathogen *Vibrio coralliilyticus* Isme J 2012 15 4 835 846 10.1038/ismej.2011.154 22158392 PMC3309362

[B6] Maurelli AT Sansonetti PJ Identification of a chromosomal gene controlling temperature-regulated expression of *Shigella* virulence P Natl Acad Sci USA 1988 15 8 2820 2824 10.1073/pnas.85.8.2820 PMC280091 3282241

[B7] Elphinstone JG Allen C Prior P Hayward AC The current Bacterial Wilt situation: A global overview Bacterial Wilt Disease and the Ralstonia solanacearum Species Complex USA 2005 St. Paul, Minnesota: APS Press 9 28

[B8] Buddenhagen I Sequeira L Kelman A Designation of races in *Pseudomonas solanacearum* Phytopathol 1962 15 8 726 1962

[B9] Hayward AC Characteristics of *Pseudomonas solanacearum* J Appl Microbiol 1964 15 2 265 277

[B10] Fegan M Prior P Allen PP C, Hayward AC How complex is the “*Ralstonia solanacearum* species complex” Bacterial Wilt Disease and the Ralstonia Solanacearum Species Complex 2005 Madison, WI: APS Press 449 462

[B11] Thurston HD Bacterial wilt of potatoes in Colombia Amer Potato J 1963 15 11 381 390 10.1007/BF02849446

[B12] Janse JD van den Beld HE Elphinstone J Simpkins S Tjou-Tam-Sin NNA van Vaerenbergh J Introduction to Europe of *Ralstonia solanacearum* biovar 2, race 3 in *Pelargonium zonale* cuttings J Plant Pathol 2004 15 2 147 155

[B13] Ciampi L Sequeira L Influence of temperature on virulence of race 3 strains of *Pseudomonas solanacearum* Am Potato J 1980 15 7 307 317 10.1007/BF02854025

[B14] Lambert CD Agricultural bioterrorism protection act of 2002: possession, use, and transfer of biological; agents and toxins; interim and final rule. (7 CFR Part 331) Fed Regist 2002 15 76908 76938

[B15] Duan Y Norman D Gabriel D Distribution and sequence analysis of putative determinants of race, biovar and cold tolerance factors of *Ralstonia solanacearum* Phytopathol 2005 15 6 S26

[B16] Denny TP Milling AS Bhakta VG Allen C *Ralstonia solanacearum* race 3 biovar 2 strains are not uniquely cold tolerant *in vitro* Phytopathol 2007 15 7 S28

[B17] Bocsanczy AM Achenbach UC Mangravita-Novo A Yuen JM Norman DJ Comparative effect of low temperature on virulence and twitching motility of *Ralstonia solanacearum* strains present in Florida Phytopathol 2012 15 2 185 194 10.1094/PHYTO-05-11-0145 21936660

[B18] Beranova-Giorgianni S Proteome analysis by two-dimensional gel electrophoresis and mass spectrometry: strengths and limitations Trac-Trend Anal Chem 2003 15 5 273 10.1016/S0165-9936(03)00508-9

[B19] Giglio MG Collmer CW Lomax J Ireland A Special issue: gene ontology for microbiologists applying the gene ontology in microbial annotation Trends Microbiol 2009 15 7 262 268 10.1016/j.tim.2009.04.003 19577473

[B20] Carbon S Ireland A Mungall CJ Shu S Marshall B Lewis S AmiGO: online access to ontology and annotation data Bioinformatics 2009 15 2 288 289 10.1093/bioinformatics/btn615 19033274 PMC2639003

[B21] Flores-Cruz Z Allen C *Ralstonia solanacearum* encounters an oxidative environment during tomato infection Mol Plant Microbe In 2009 15 7 773 782 10.1094/MPMI-22-7-0773 19522559

[B22] Slater S Houmiel KL Tran M Mitsky TA Taylor NB Padgette SR Gruys KJ Multiple beta-ketothiolases mediate poly(beta-hydroxyalkanoate) copolymer synthesis in *Ralstonia eutropha* J Bacteriol 1998 15 8 1979 1987 9555876 10.1128/jb.180.8.1979-1987.1998 PMC107120

[B23] Schlegel HG Vonbartheld R Gottschalk G Formation and utilization of poly-beta-hydroxybutyric acid by Knallgas bacteria (*Hydrogenomonas*) Nature 1961 15 478 463 13747776 10.1038/191463a0

[B24] Handrick R Reinhardt S Kimmig P Jendrossek D The “intracellular” poly(3-hydroxybutyrate) (PHB) depolymerase of *Rhodospirillum rubrum* is a periplasm-located protein with specificity for native PHB and with structural similarity to extracellular PHB depolymerases J Bacteriol 2004 15 21 7243 7253 10.1128/JB.186.21.7243-7253.2004 15489436 PMC523223

[B25] Hennequin C Collignon A Karjalainen T Analysis of expression of GroEL (Hsp60) of *Clostridium difficile* in response to stress Microb Pathogenesis 2001 15 5 255 260 10.1006/mpat.2001.0468 11710845

[B26] Thomas JG Baneyx F ClpB and HtpG facilitate *de novo* protein folding in stressed *Escherichia coli* cells Mol Microbiol 2000 15 6 1360 1370 10931286 10.1046/j.1365-2958.2000.01951.x

[B27] Hernandez-Romero D Solano F Sanchez-Amat A Polyphenol oxidase activity expression in *Ralstonia solanacearum* Appl Environ Microbiol 2005 15 11 6808 6815 10.1128/AEM.71.11.6808-6815.2005 16269713 PMC1287666

[B28] Colburn-Clifford JM Scherf JM Allen C *Ralstonia solanacearum* Dps contributes to oxidative stress tolerance and to colonization of and virulence on tomato plants Appl Environ Microbiol 2010 15 22 7392 7399 10.1128/AEM.01742-10 20870795 PMC2976212

[B29] Xu XQ Pan SQ An agrobacterium catalase is a virulence factor involved in tumorigenesis Mol Microbiol 2000 15 2 407 414 10.1046/j.1365-2958.2000.01709.x 10652101

[B30] Liu H Kang Y Genin S Schell MA Denny TP Twitching motility of *Ralstonia solanacearum* requires a type IV pilus system Microbiol (Reading) 2001 15 12 3215 3229 10.1099/00221287-147-12-3215 11739754

[B31] Kang Y Liu H Genin S Schell MA Denny TP *Ralstonia solanacearum* requires type 4 pili to adhere to multiple surfaces and for natural transformation and virulence Mol Microbiol 2002 15 2 427 437 10.1046/j.1365-2958.2002.03187.x 12406219

[B32] Mougous JD Cuff ME Raunser S Shen A Zhou M Gifford CA Goodman AL Joachimiak G Ordonez CL Lory S Waltz T Joachimiak A Mekalanos JJ A virulence locus of *Pseudomonas aeruginosa* encodes a protein secretion apparatus Science 2006 15 5779 1526 1530 10.1126/science.1128393 16763151 PMC2800167

[B33] Pukatzki S Ma AT Sturtevant D Krastins B Sarracino D Nelson WC Heidelberg JF Mekalanos JJ Identification of a conserved bacterial protein secretion system in *Vibrio cholerae* using the *Dictyostelium* host model system P Natl Acad Sci USA 2006 15 5 1528 1533 10.1073/pnas.0510322103 PMC1345711 16432199

[B34] Sarris PF Skandalis N Kokkinidis M Panopoulos NJ *In silico* analysis reveals multiple putative type VI secretion systems and effector proteins in *Pseudomonas syringae* pathovars Mol Plant Pathol 2010 15 6 795 804 21091602 10.1111/j.1364-3703.2010.00644.x PMC6640432

[B35] Zheng J Leung KY Dissection of a type VI secretion system in *Edwardsiella tarda* Mol Microbiol 2007 15 5 1192 1206 10.1111/j.1365-2958.2007.05993.x 17986187

[B36] Haapalainen M Mosorin H Dorati F Wu RF Roine E Taira S Nissinen R Mattinen L Jackson R Pirhonen M Lin NC Hcp2, a secreted protein of the phytopathogen *Pseudomonas syringae* pv. tomato DC3000, is required for fitness for competition against bacteria and yeasts J Bacteriol 2012 15 18 4810 4822 10.1128/JB.00611-12 22753062 PMC3430304

[B37] Wu HY Chung PC Shih HW Wen SR Lai EM Secretome analysis uncovers an hcp-family protein secreted via a type VI secretion system in *Agrobacterium tumefaciens* J Bacteriol 2008 15 8 2841 2850 10.1128/JB.01775-07 18263727 PMC2293243

[B38] Bingle LEH Bailey CM Pallen MJ Type VI secretion: a beginner’s guide Curr Opin Microbiol 2008 15 1 3 8 10.1016/j.mib.2008.01.006 18289922

[B39] Bladergroen MR Badelt K Spaink HP Infection-blocking genes of a symbiotic *Rhizobium leguminosarum* strain that are involved in temperature-dependent protein secretion Mol Plant Microbe In 2003 15 1 53 64 10.1094/MPMI.2003.16.1.53 12580282

[B40] da Silva FG Shen YW Dardick C Burdman S Yadav RC de Leon AL Ronald PC Bacterial genes involved in type I secretion and sulfation are required to elicit the rice Xa21-mediated innate immune response Mol Plant Microbe In 2004 15 6 593 601 10.1094/MPMI.2004.17.6.593 15195942

[B41] Lomovskaya O Lewis K Emr, an *Escherichia coli* locus for multidrug resistance P Natl Acad Sci USA 1992 15 19 8938 8942 10.1073/pnas.89.19.8938 PMC50039 1409590

[B42] Colmer JA Fralick JA Hamood AN Isolation and characterization of a putative multidrug resistance pump from *Vibrio cholerae* Mol Microbiol 1998 15 1 63 72 10.1046/j.1365-2958.1998.00657.x 9466256

[B43] Xu J Zheng HJ Liu L Pan ZC Prior P Tang B Xu JS Zhang H Tian Q Zhang LQ Feng J Complete genome sequence of the plant pathogen *Ralstonia solanacearum* strain Po82 J Bacteriol 2011 15 16 4261 4262 10.1128/JB.05384-11 21685279 PMC3147697

[B44] Stevens P van Overbeek LS van Elsas JD *Ralstonia solanacearum* Delta PGI-1 strain KZR-5 is affected in growth, response to cold stress and invasion of tomato Microb Ecol 2011 15 1 101 112 10.1007/s00248-010-9728-0 20717661 PMC3011089

[B45] Liu HL Zhang SP Schell MA Denny TP Pyramiding, unmarked deletions in *Ralstonia solanacearum* shows that secreted proteins in addition to plant cell-wall-degrading enzymes contribute to virulence Mol Plant Microbe In 2005 15 12 1296 1305 10.1094/MPMI-18-1296 16478049

[B46] Boucher CA Gough CL Arlat M Molecular genetics of pathogenicity determinants of *Pseudomonas solanacearum* with special emphasis on *hrp* genes Annu Rev Phytopathol 1992 15 443 461 10.1146/annurev.py.30.090192.002303

[B47] Denny TP Baek SR Genetic evidence that extracellular polysaccharide is a virulence factor of *Pseudomonas solanacearum* Mol Plant Microbe In 1991 15 2 198 206 10.1094/MPMI-4-198

[B48] Brencic A Winans SC Detection of and response to signals involved in host-microbe interactions by plant-associated bacteria Microbiol Mol Biol R 2005 15 1 155 10.1128/MMBR.69.1.155-194.2005 PMC1082791 15755957

[B49] Yao J Allen C Chemotaxis is required for virulence and competitive fitness of the bacterial wilt pathogen *Ralstonia solanacearum* J Bacteriol 2006 15 10 3697 3708 10.1128/JB.188.10.3697-3708.2006 16672623 PMC1482862

[B50] Pukatzki S Ma AT Revel AT Sturtevant D Mekalanos JJ Type VI secretion system translocates a phage tail spike-like protein into target cells where it cross-links actin P Natl Acad Sci USA 2007 15 39 15508 15513 10.1073/pnas.0706532104 PMC2000545 17873062

[B51] Schell MA Control of virulence and pathogenicity genes of *Ralstonia solanacearum* by an elaborate sensory network Annu Rev Phytopathol 2000 15 263 292 10.1146/annurev.phyto.38.1.263 11701844

[B52] Brumbley SM Carney BF Denny TP Phenotype conversion in *Pseudomonas solanacearum* due to spontaneous inactivation of PhcA, a putative LysR transcriptional regulator J Bacteriol 1993 15 17 5477 5487 8366033 10.1128/jb.175.17.5477-5487.1993 PMC206604

[B53] Huang JZ Carney BF Denny TP Weissinger AK Schell MA A complex network regulates expression of *eps* and other virulence genes of *Pseudomonas solanacearum* J Bacteriol 1995 15 5 1259 1267 7868600 10.1128/jb.177.5.1259-1267.1995 PMC176732

[B54] Nissinen RM Ytterberg AJ Bogdanove AJ KJVANW Beer SV Analyses of the secretomes of *Erwinia amylovora* and selected hrp mutants reveal novel type III secreted proteins and an effect of HrpJ on extracellular harpin levels Mol Plant Pathol 2007 15 1 55 67 10.1111/j.1364-3703.2006.00370.x 20507478

[B55] Keller A Nesvizhskii AI Kolker E Aebersold R Empirical statistical model to estimate the accuracy of peptide identifications made by MS/MS and database search Anal Chem 2002 15 20 5383 5392 10.1021/ac025747h 12403597

[B56] Nesvizhskii AI Keller A Kolker E Aebersold R A statistical model for identifying proteins by tandem mass spectrometry Anal Chem 2003 15 17 4646 4658 10.1021/ac0341261 14632076

[B57] Jahn CE Charkowski AO Willis DK Evaluation of isolation methods and RNA integrity for bacterial RNA quantitation J Microbiol Meth 2008 15 2 318 324 10.1016/j.mimet.2008.07.004 18674572

