# Supplementary material for: Proteomic comparison of Ralstonia solanacearum strains reveals temperature dependent virulence factors
Source: BMC Genomics. 2014 Apr 12;15:280. doi: 10.1186/1471-2164-15-280 (PMC4023598; doi:10.1186/1471-2164-15-280)
Supplement: Additional file 4 — Gel images for comparative experiment of two strains to illustrate the process of gel comparisons. Includes comparative gels and images with spots numbered and normalized for GMI1000 and P673 comparative gels of cell-associated proteins. [file 1471-2164-15-280-S4.pptx]

## Slide 1
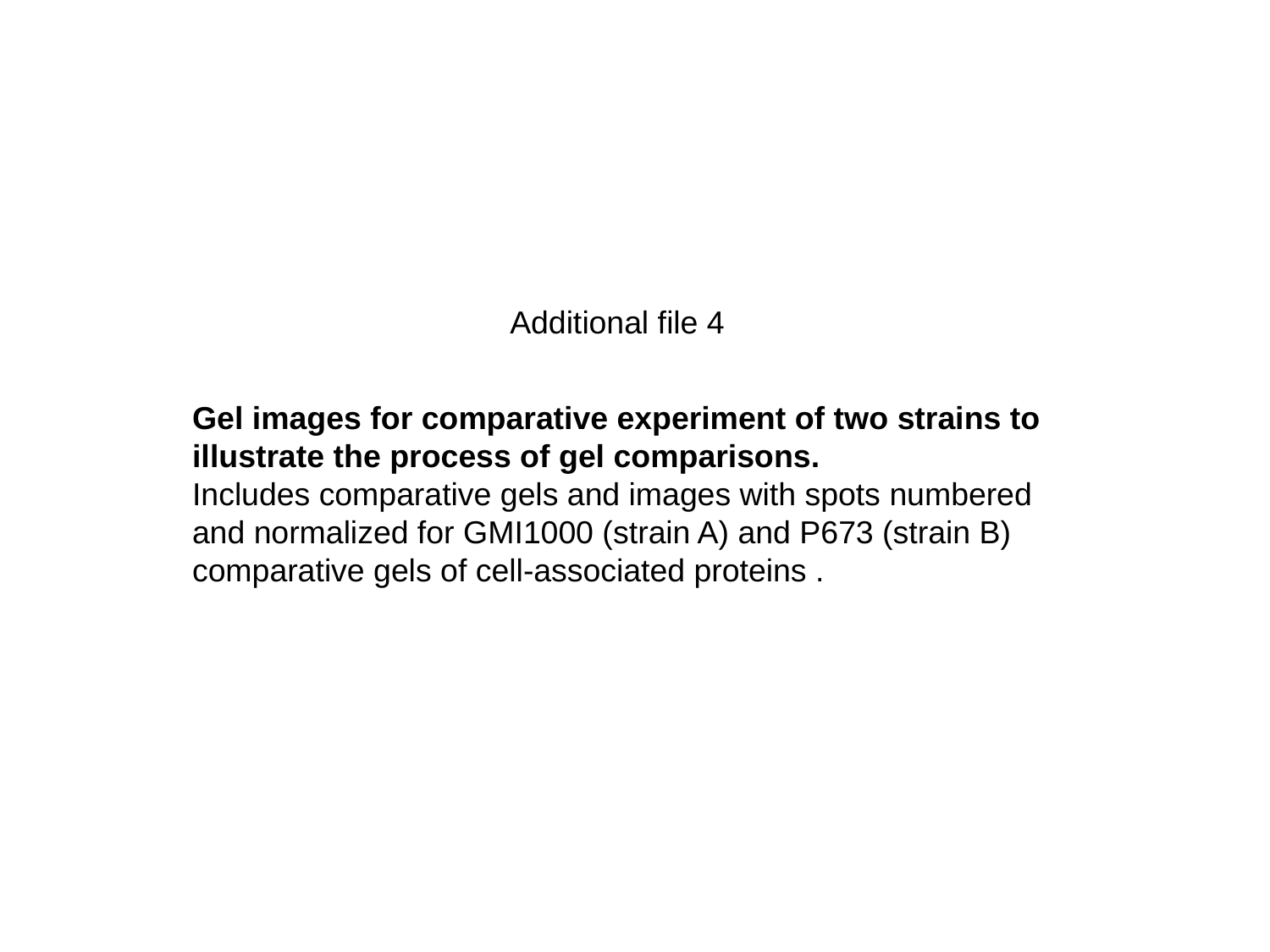

Additional file 4
Gel images for comparative experiment of two strains to illustrate the process of gel comparisons.
Includes comparative gels and images with spots numbered and normalized for GMI1000 (strain A) and P673 (strain B) comparative gels of cell-associated proteins .

## Slide 2
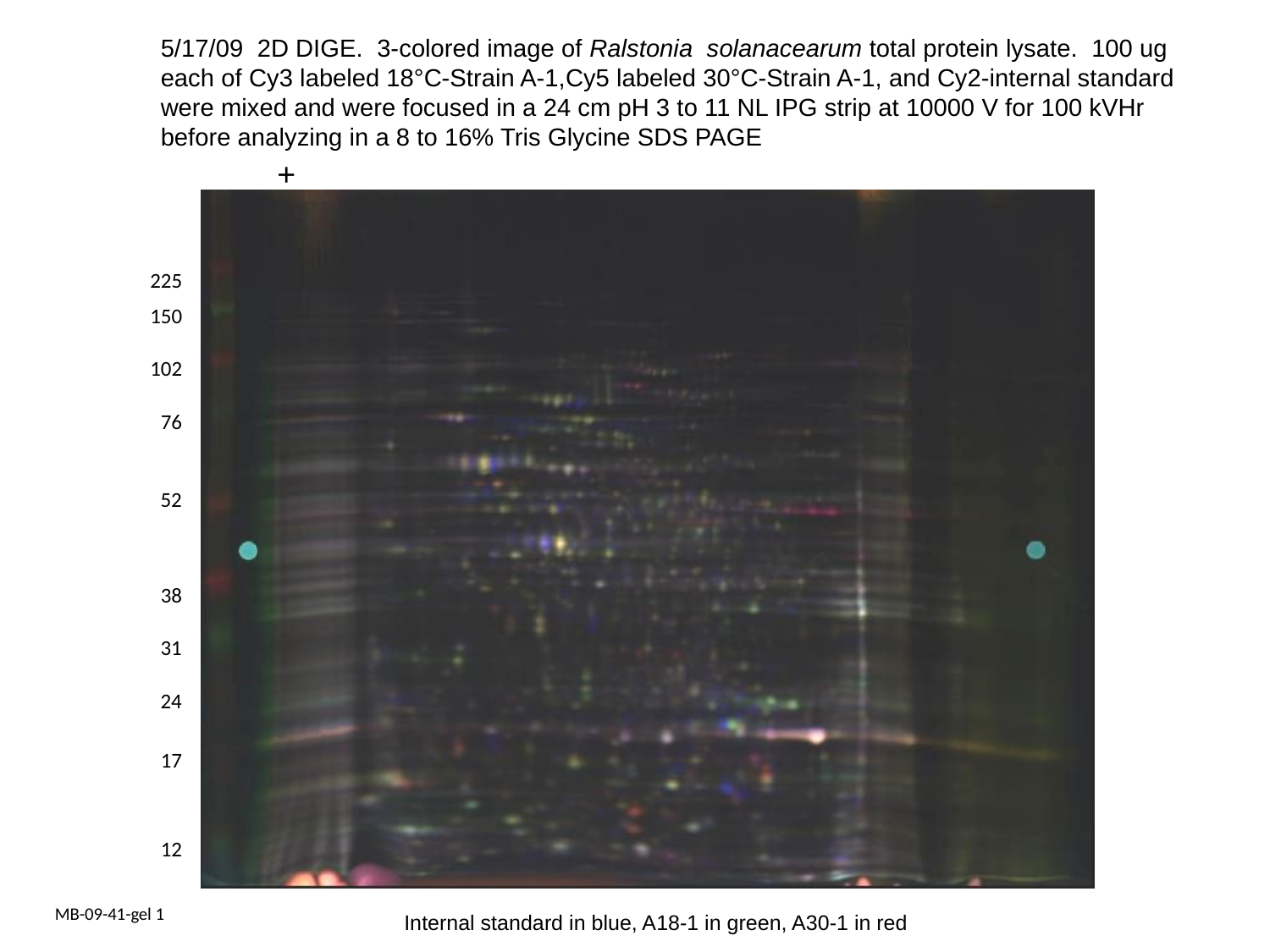

5/17/09 2D DIGE. 3-colored image of Ralstonia solanacearum total protein lysate. 100 ug each of Cy3 labeled 18°C-Strain A-1,Cy5 labeled 30°C-Strain A-1, and Cy2-internal standard were mixed and were focused in a 24 cm pH 3 to 11 NL IPG strip at 10000 V for 100 kVHr before analyzing in a 8 to 16% Tris Glycine SDS PAGE
+
225
150
102
76
52
38
31
24
17
12
MB-09-41-gel 1
Internal standard in blue, A18-1 in green, A30-1 in red

## Slide 3
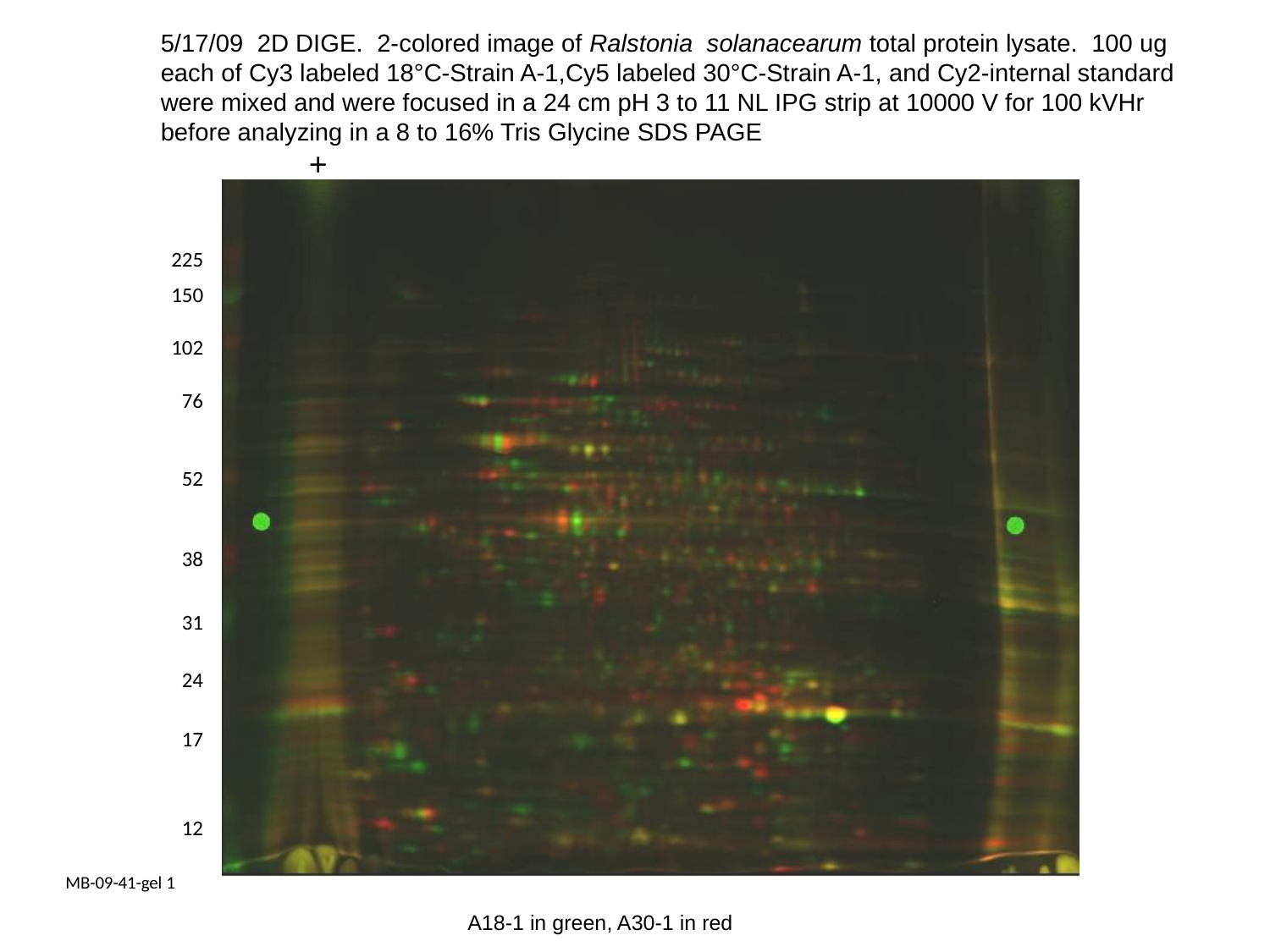

5/17/09 2D DIGE. 2-colored image of Ralstonia solanacearum total protein lysate. 100 ug each of Cy3 labeled 18°C-Strain A-1,Cy5 labeled 30°C-Strain A-1, and Cy2-internal standard were mixed and were focused in a 24 cm pH 3 to 11 NL IPG strip at 10000 V for 100 kVHr before analyzing in a 8 to 16% Tris Glycine SDS PAGE
+
225
150
102
76
52
38
31
24
17
12
MB-09-41-gel 1
A18-1 in green, A30-1 in red

## Slide 4
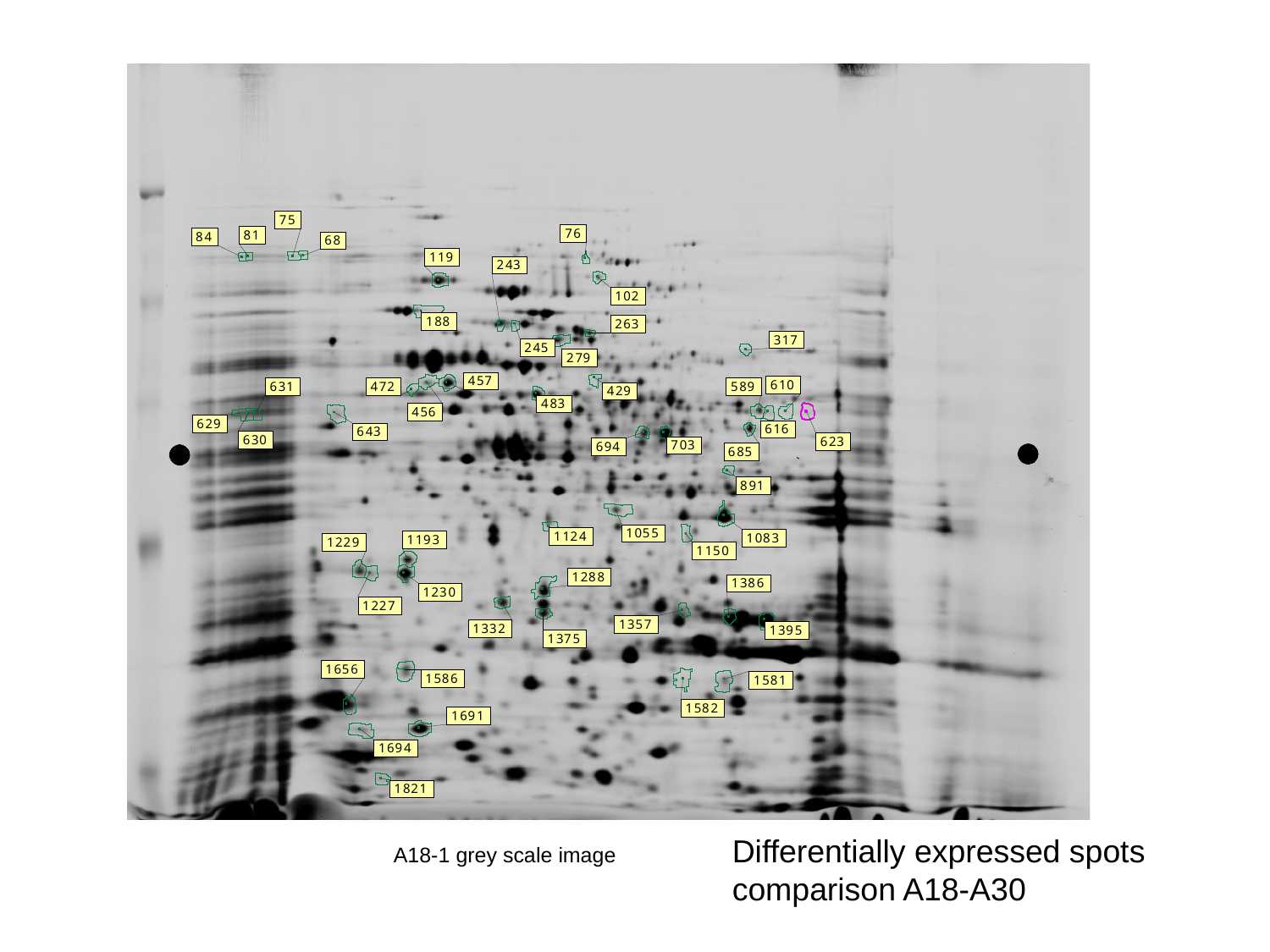

Differentially expressed spots comparison A18-A30
A18-1 grey scale image

## Slide 5
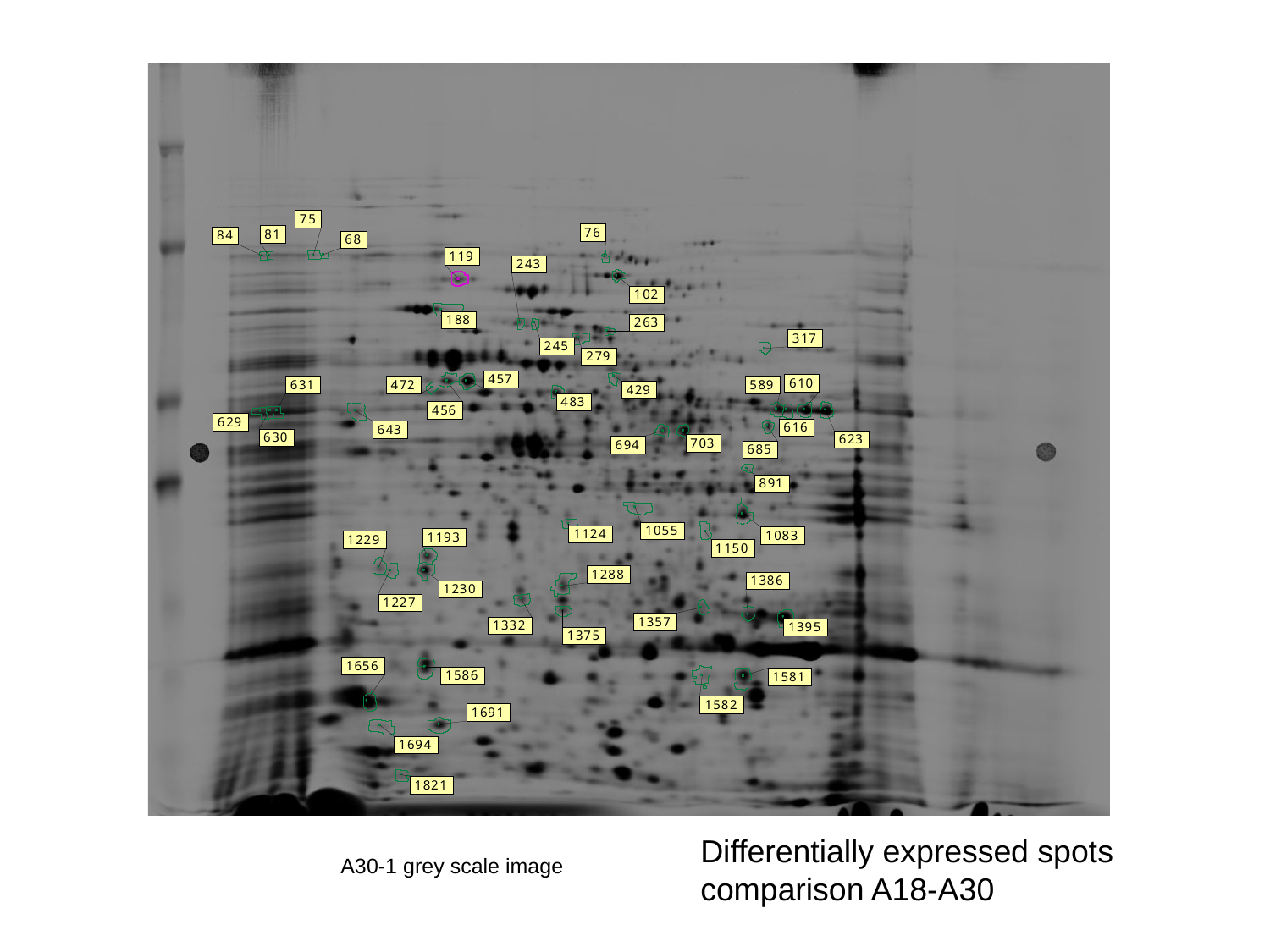

Differentially expressed spots comparison A18-A30
A30-1 grey scale image

## Slide 6
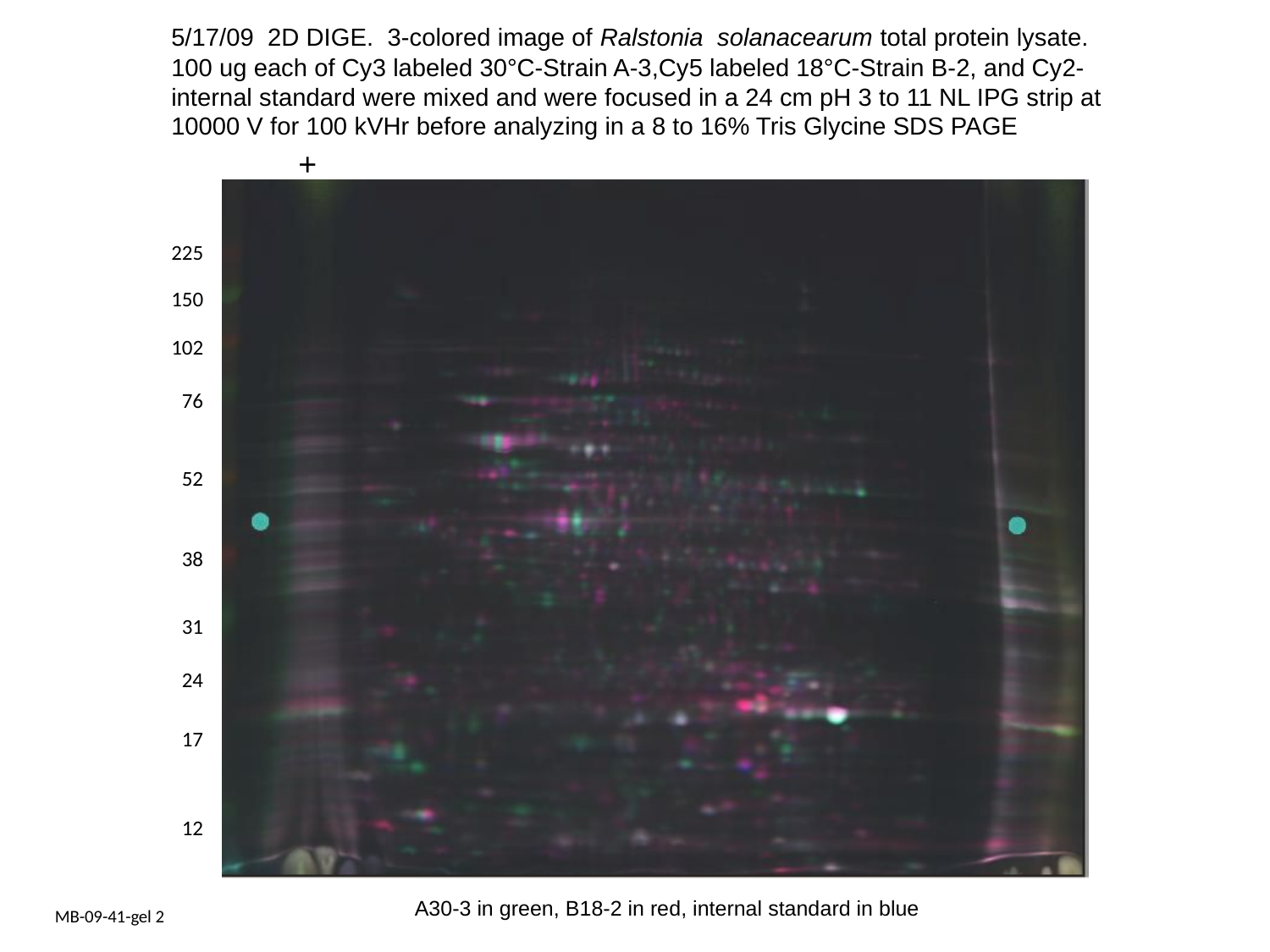

5/17/09 2D DIGE. 3-colored image of Ralstonia solanacearum total protein lysate. 100 ug each of Cy3 labeled 30°C-Strain A-3,Cy5 labeled 18°C-Strain B-2, and Cy2-internal standard were mixed and were focused in a 24 cm pH 3 to 11 NL IPG strip at 10000 V for 100 kVHr before analyzing in a 8 to 16% Tris Glycine SDS PAGE
+
225
150
102
76
52
38
31
24
17
12
A30-3 in green, B18-2 in red, internal standard in blue
MB-09-41-gel 2

## Slide 7
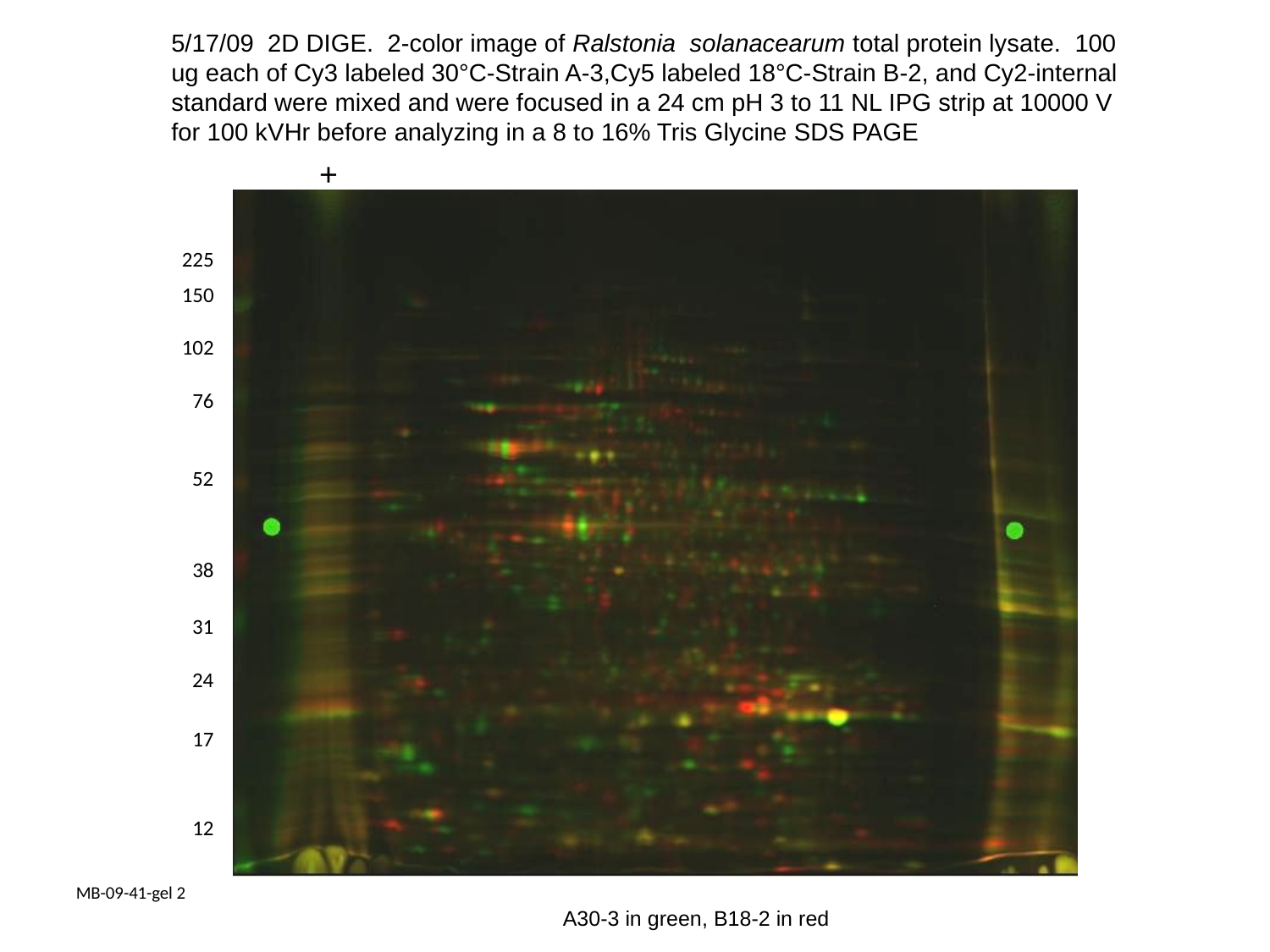

5/17/09 2D DIGE. 2-color image of Ralstonia solanacearum total protein lysate. 100 ug each of Cy3 labeled 30°C-Strain A-3,Cy5 labeled 18°C-Strain B-2, and Cy2-internal standard were mixed and were focused in a 24 cm pH 3 to 11 NL IPG strip at 10000 V for 100 kVHr before analyzing in a 8 to 16% Tris Glycine SDS PAGE
+
225
150
102
76
52
38
31
24
17
12
MB-09-41-gel 2
A30-3 in green, B18-2 in red

## Slide 8
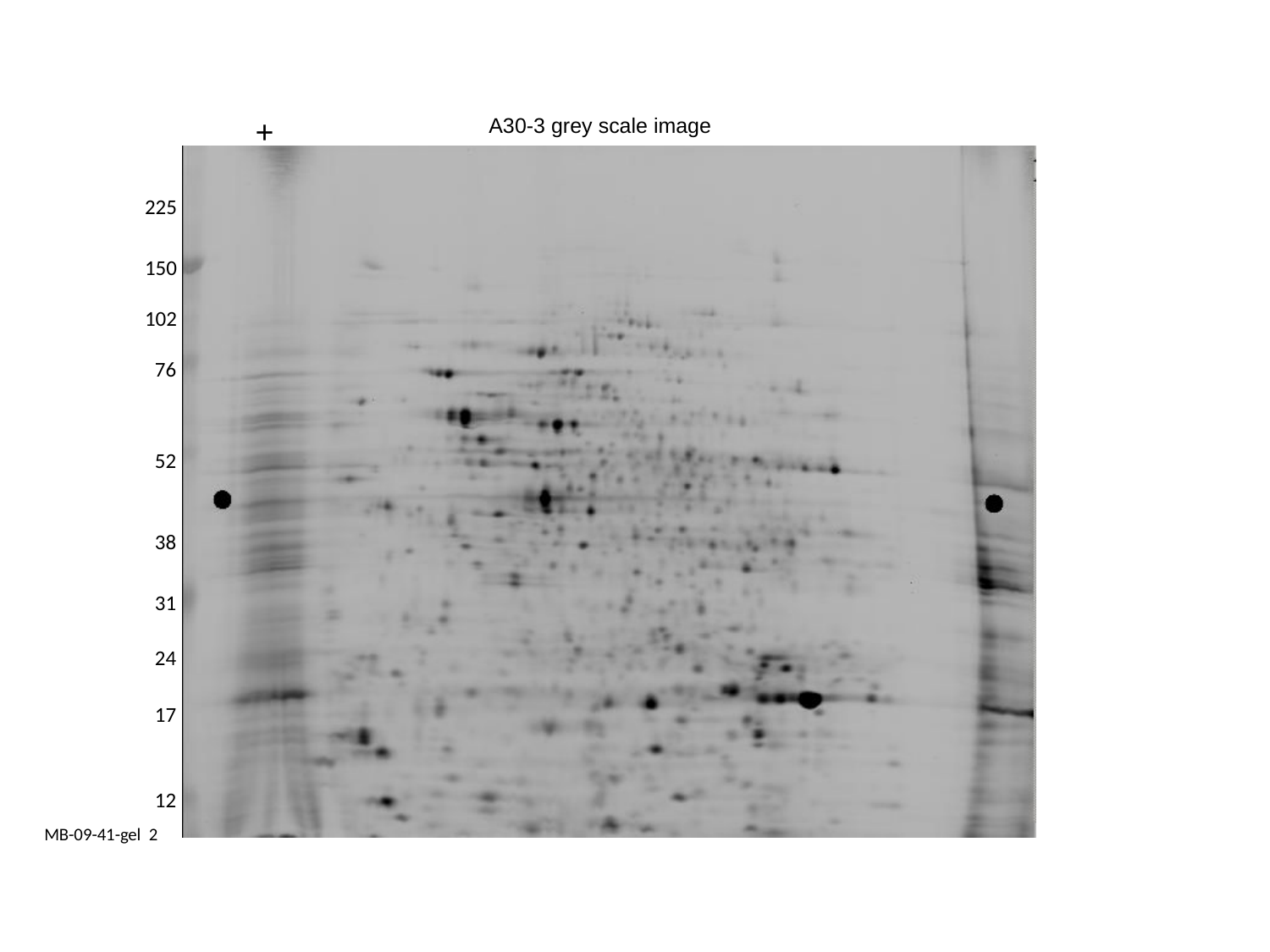

+
A30-3 grey scale image
225
150
102
76
52
38
31
24
17
12
MB-09-41-gel 2

## Slide 9
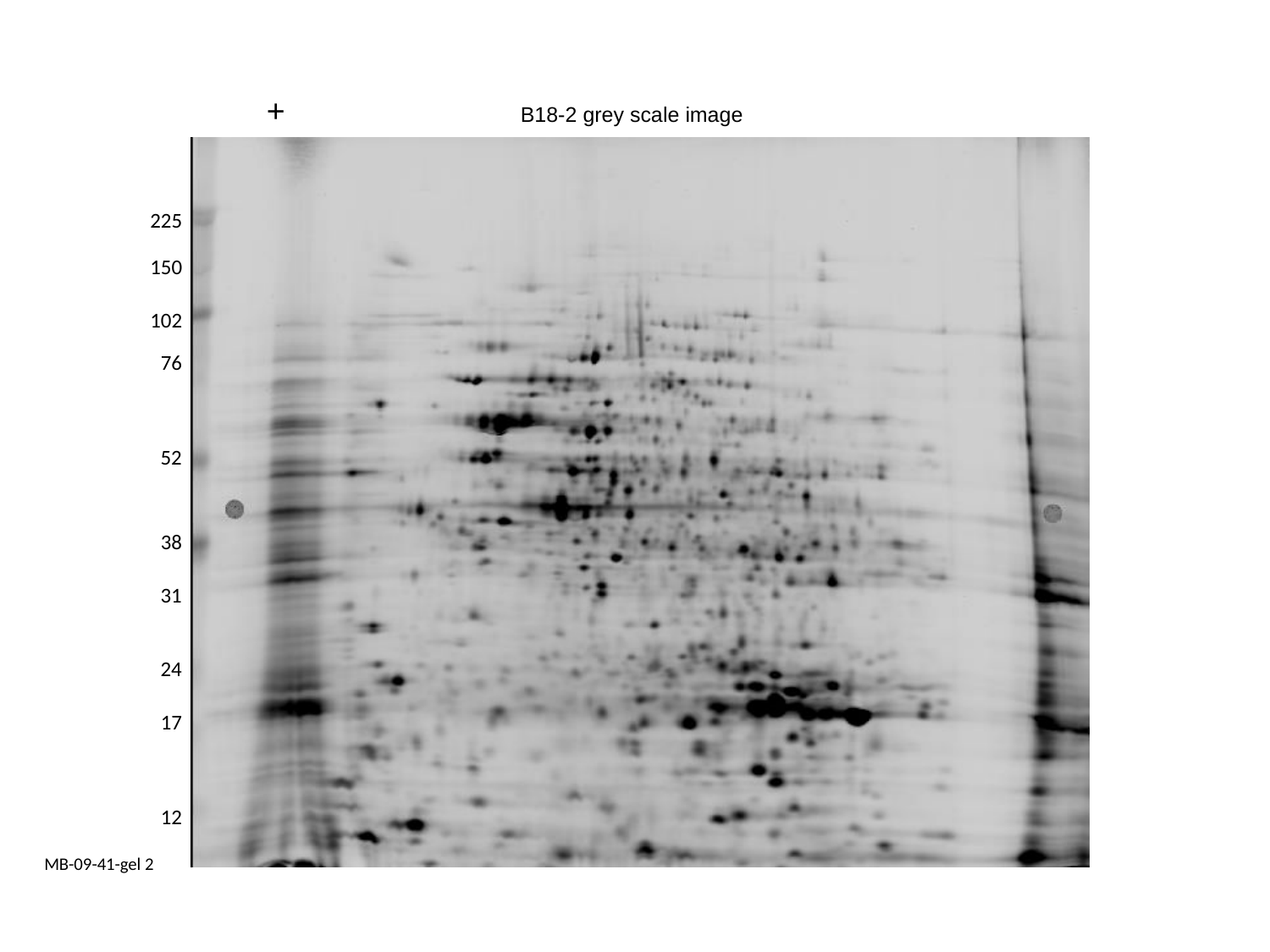

+
B18-2 grey scale image
225
150
102
76
52
38
31
24
17
12
MB-09-41-gel 2

## Slide 10
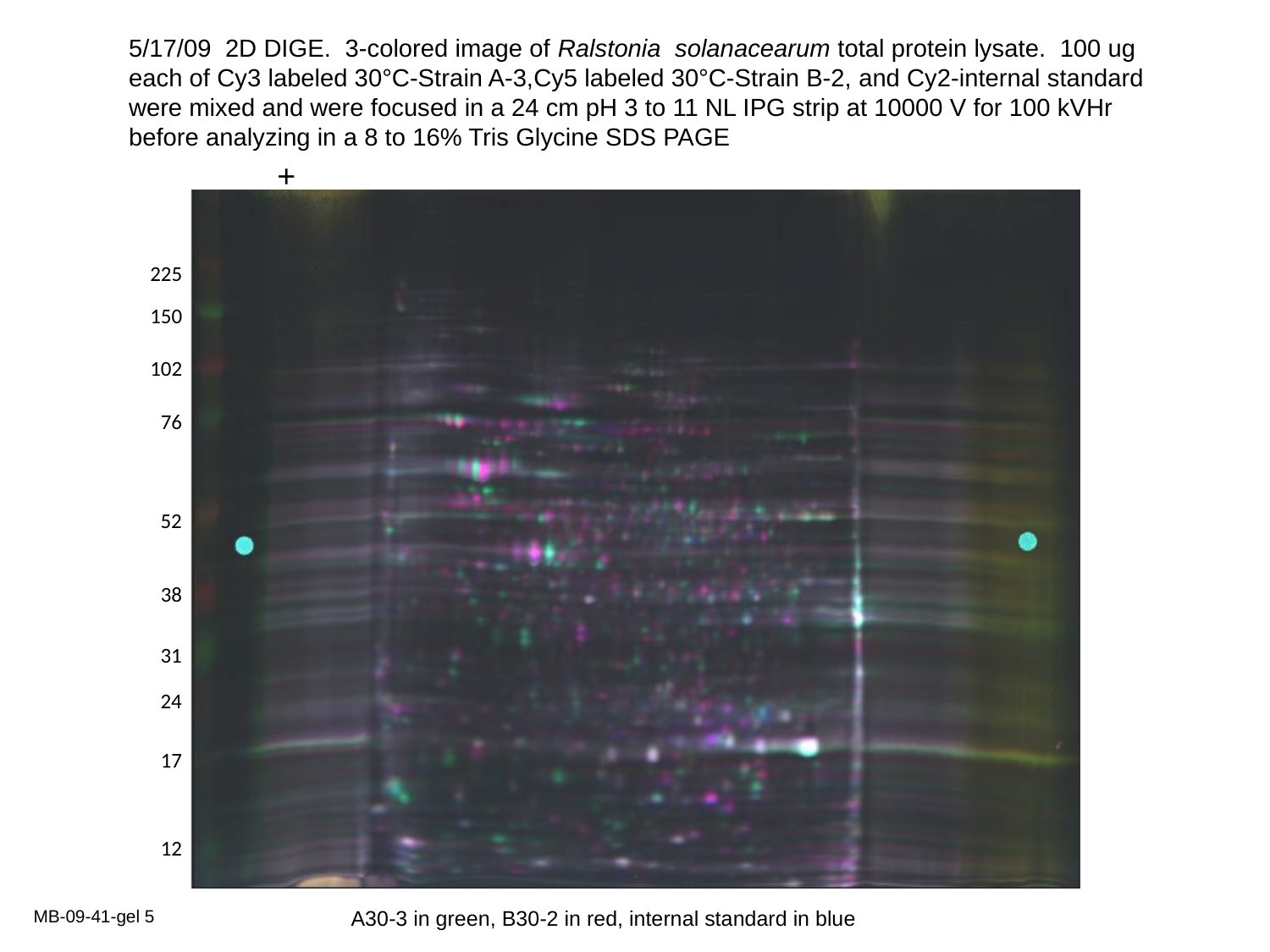

5/17/09 2D DIGE. 3-colored image of Ralstonia solanacearum total protein lysate. 100 ug each of Cy3 labeled 30°C-Strain A-3,Cy5 labeled 30°C-Strain B-2, and Cy2-internal standard were mixed and were focused in a 24 cm pH 3 to 11 NL IPG strip at 10000 V for 100 kVHr before analyzing in a 8 to 16% Tris Glycine SDS PAGE
+
225
150
102
76
52
38
31
24
17
12
MB-09-41-gel 5
A30-3 in green, B30-2 in red, internal standard in blue

## Slide 11
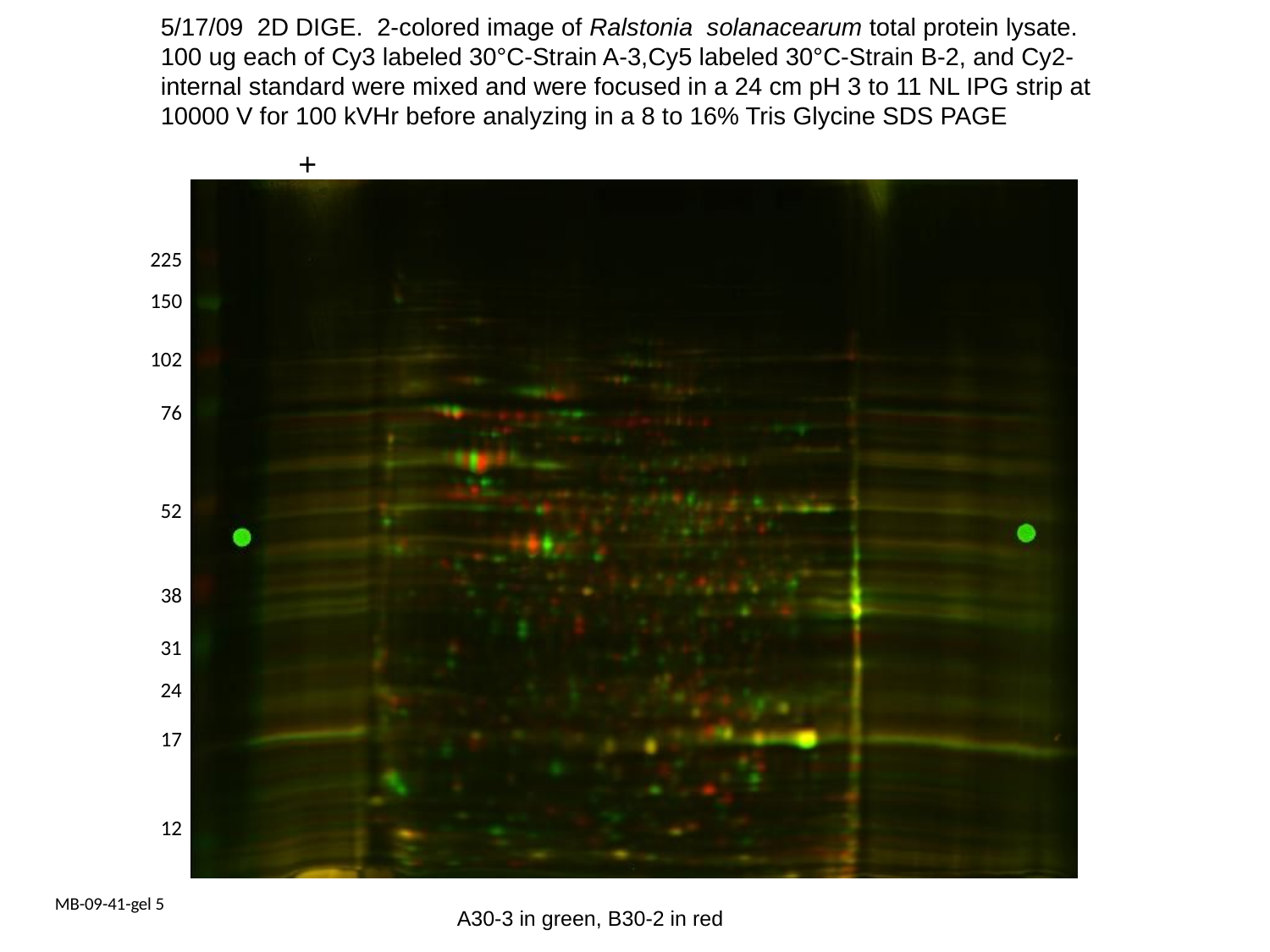

5/17/09 2D DIGE. 2-colored image of Ralstonia solanacearum total protein lysate. 100 ug each of Cy3 labeled 30°C-Strain A-3,Cy5 labeled 30°C-Strain B-2, and Cy2-internal standard were mixed and were focused in a 24 cm pH 3 to 11 NL IPG strip at 10000 V for 100 kVHr before analyzing in a 8 to 16% Tris Glycine SDS PAGE
+
225
150
102
76
52
38
31
24
17
12
MB-09-41-gel 5
A30-3 in green, B30-2 in red

## Slide 12
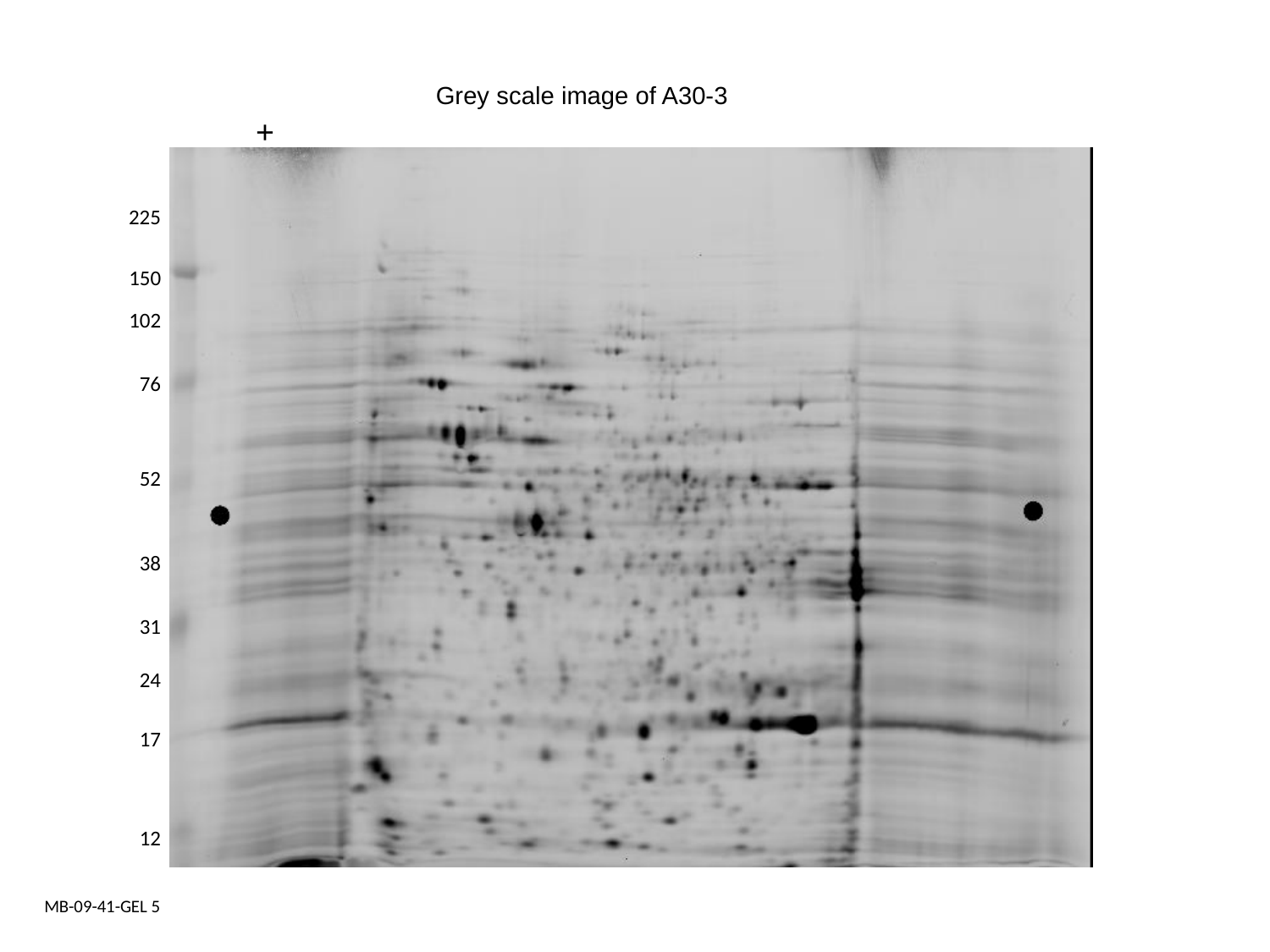

Grey scale image of A30-3
+
225
150
102
76
52
38
31
24
17
12
MB-09-41-GEL 5

## Slide 13
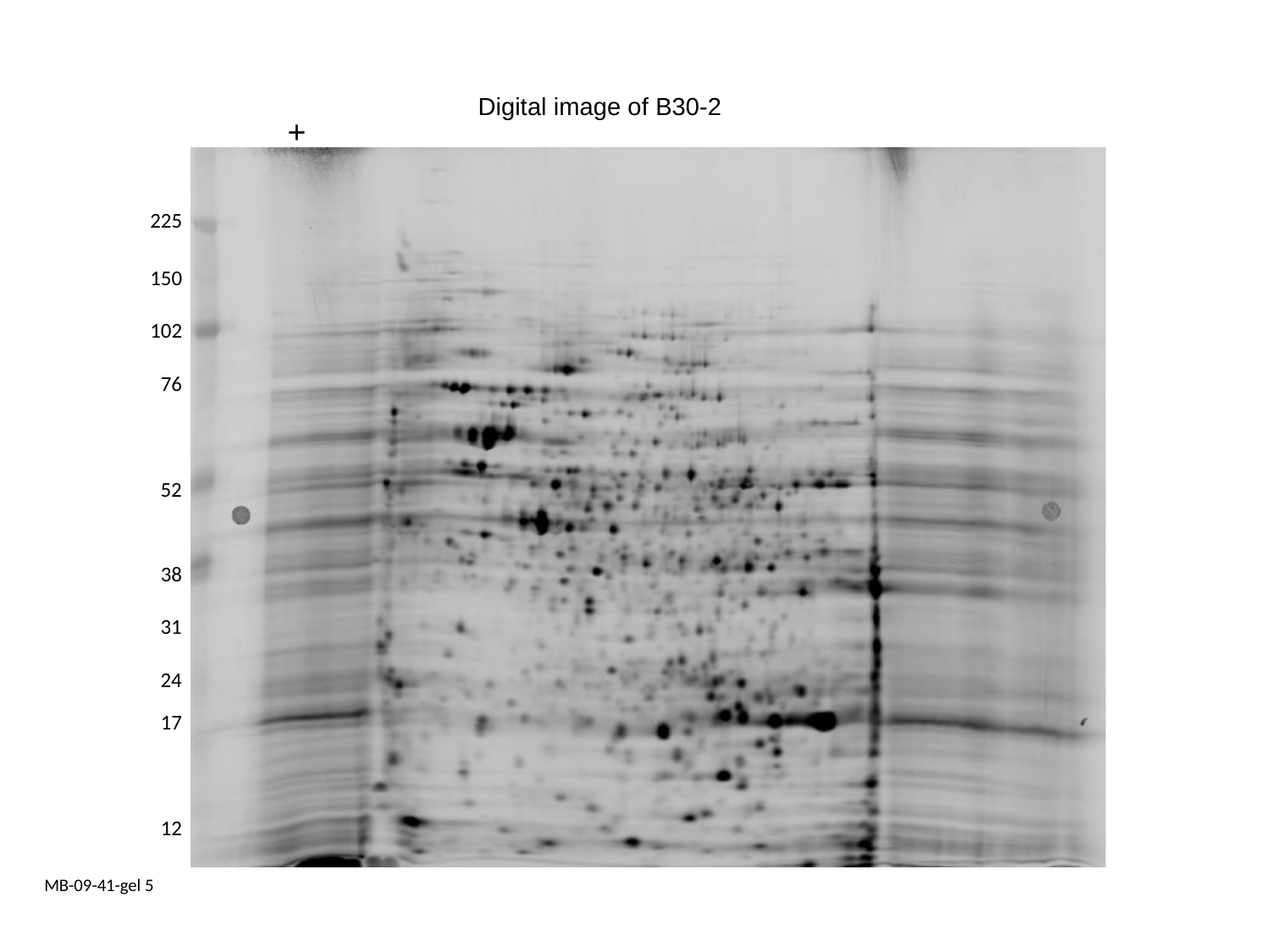

Digital image of B30-2
+
225
150
102
76
52
38
31
24
17
12
MB-09-41-gel 5

## Slide 14
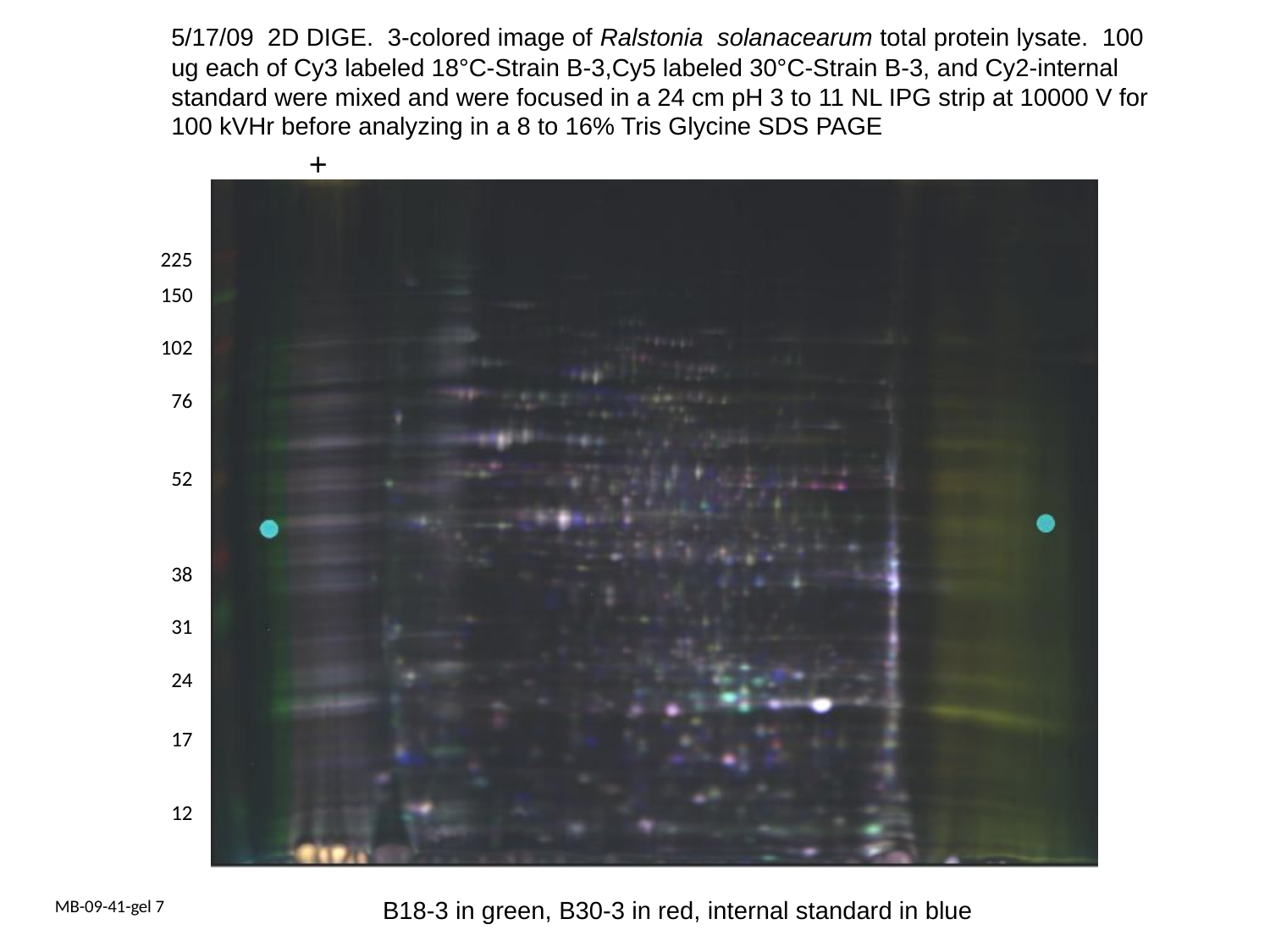

5/17/09 2D DIGE. 3-colored image of Ralstonia solanacearum total protein lysate. 100 ug each of Cy3 labeled 18°C-Strain B-3,Cy5 labeled 30°C-Strain B-3, and Cy2-internal standard were mixed and were focused in a 24 cm pH 3 to 11 NL IPG strip at 10000 V for 100 kVHr before analyzing in a 8 to 16% Tris Glycine SDS PAGE
+
225
150
102
76
52
38
31
24
17
12
MB-09-41-gel 7
B18-3 in green, B30-3 in red, internal standard in blue

## Slide 15
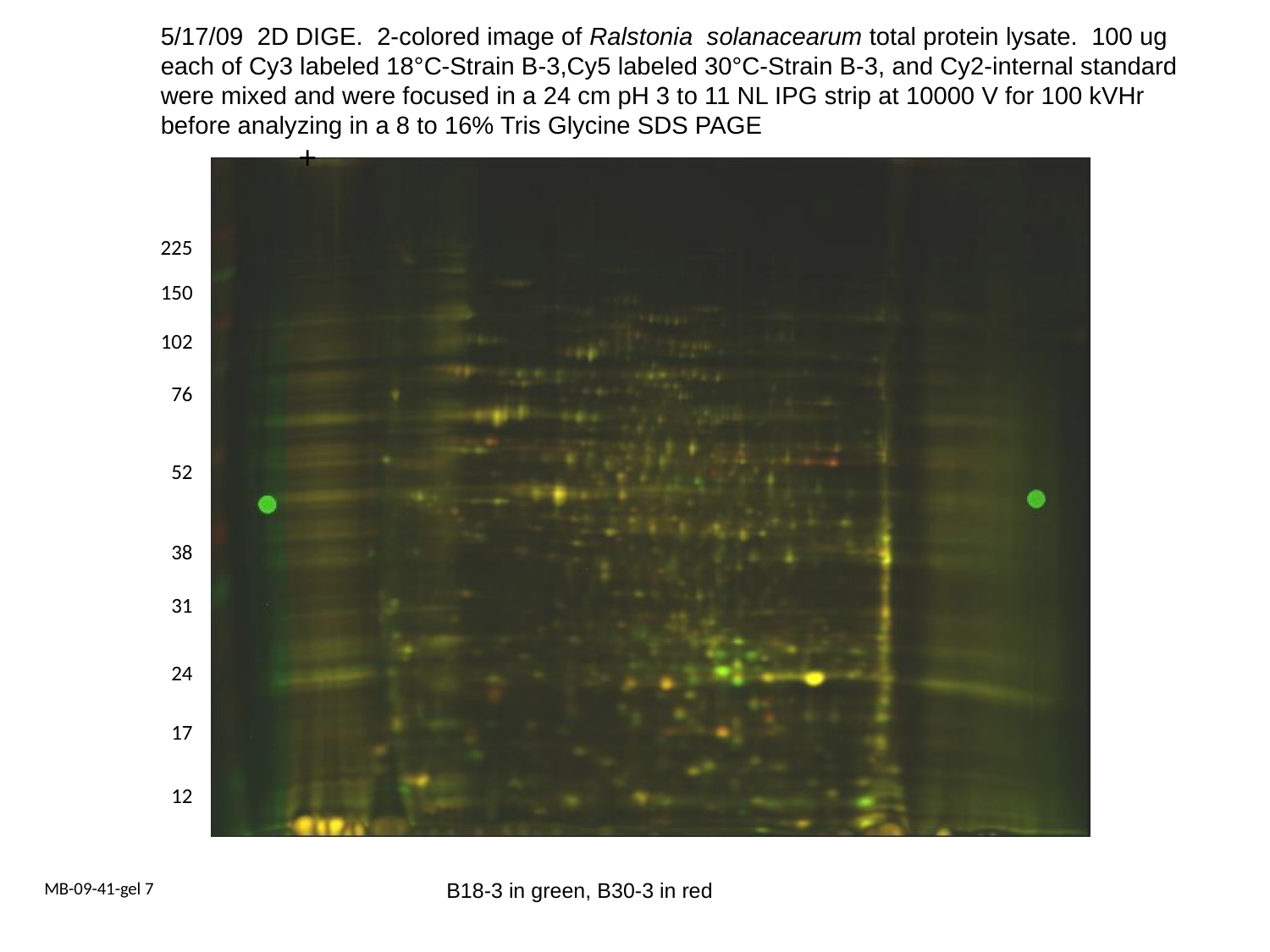

5/17/09 2D DIGE. 2-colored image of Ralstonia solanacearum total protein lysate. 100 ug each of Cy3 labeled 18°C-Strain B-3,Cy5 labeled 30°C-Strain B-3, and Cy2-internal standard were mixed and were focused in a 24 cm pH 3 to 11 NL IPG strip at 10000 V for 100 kVHr before analyzing in a 8 to 16% Tris Glycine SDS PAGE
+
225
150
102
76
52
38
31
24
17
12
MB-09-41-gel 7
B18-3 in green, B30-3 in red

## Slide 16
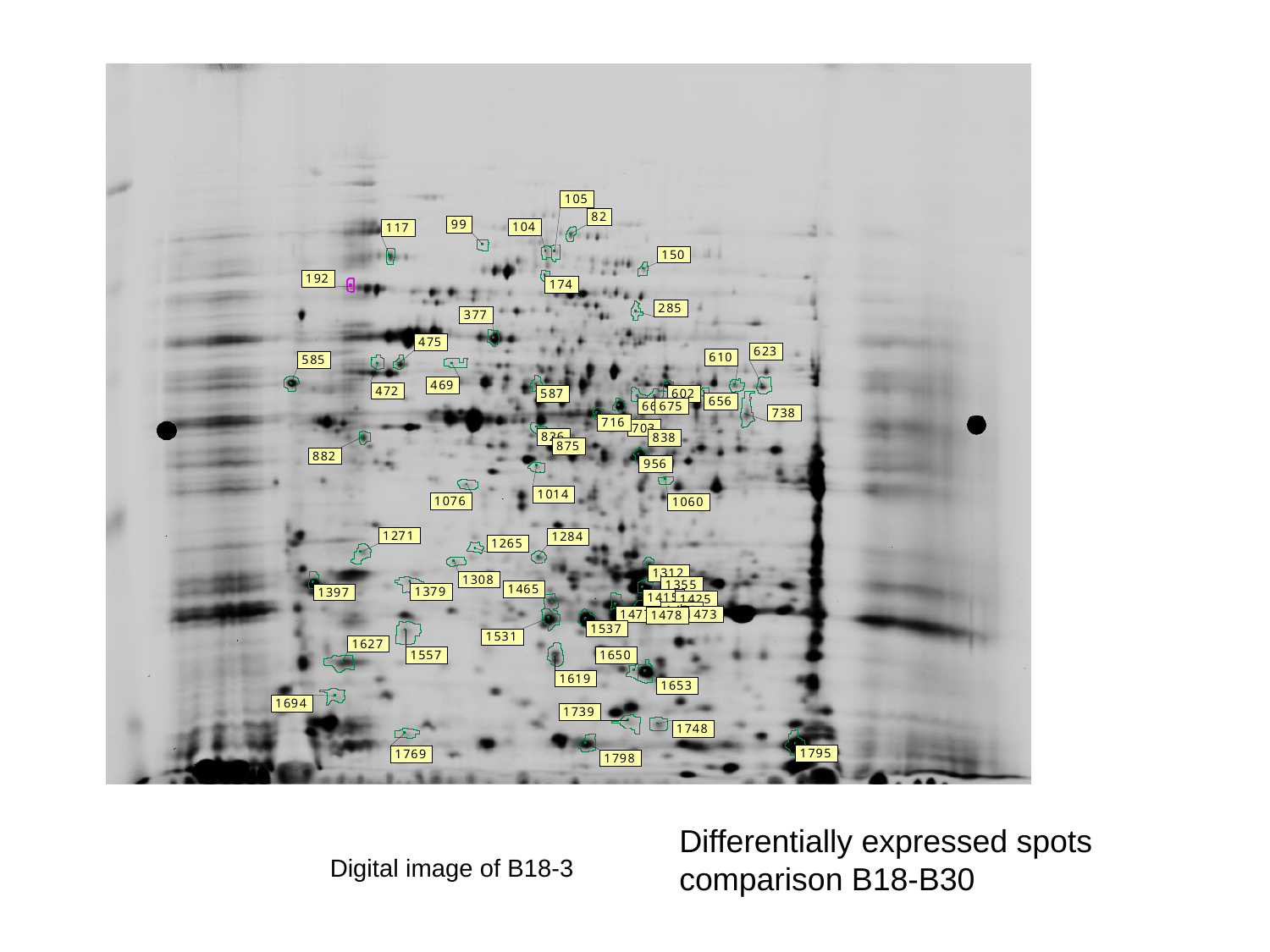

Differentially expressed spots comparison B18-B30
Digital image of B18-3

## Slide 17
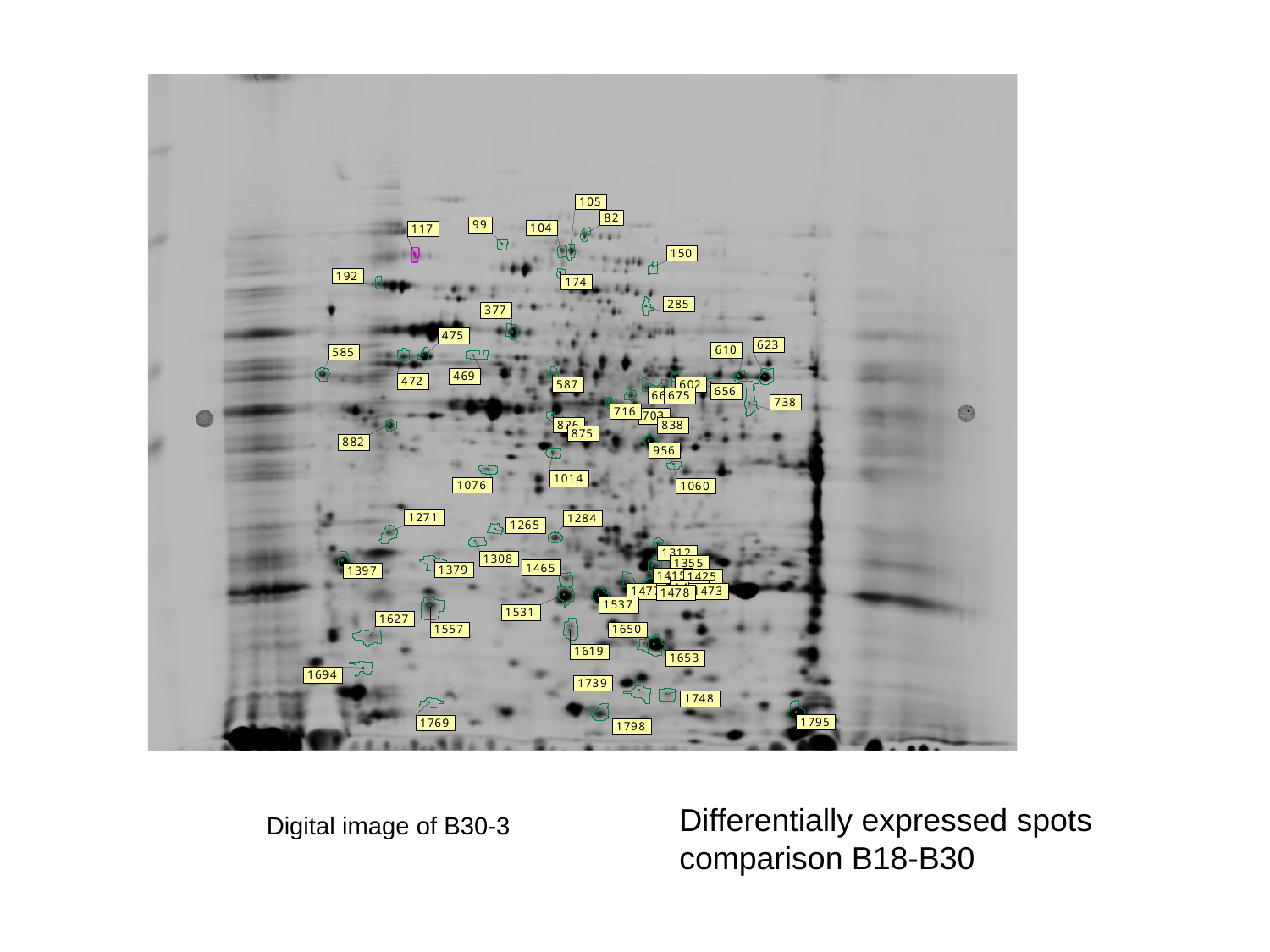

Differentially expressed spots comparison B18-B30
Digital image of B30-3
